# Toward the Photocatalytic Valorization of Lignin: Conversion of a Model Lignin Hexamer with Multiple Functionalities

**DOI:** 10.1021/acssuschemeng.2c01606

**Published:** 2022-09-02

**Authors:** Christopher W. J. Murnaghan, Nathan Skillen, Bronagh Hackett, Jack Lafferty, Peter K. J. Robertson, Gary N. Sheldrake

**Affiliations:** School of Chemistry and Chemical Engineering, Queen’s University Belfast, David Keir Building, Stranmillis Road, Belfast BT9 5AG, U.K.

**Keywords:** lignin models, photocatalysis, sustainable conversion, biomass valorization, degradation pathway

## Abstract

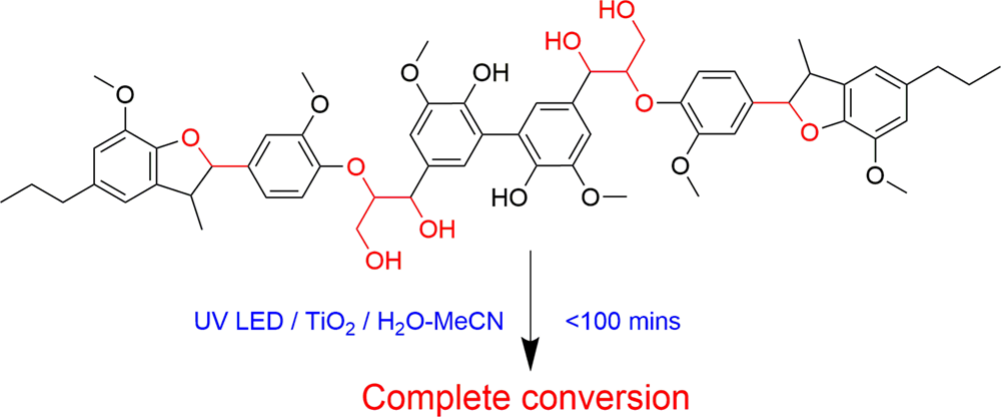

The valorization of biomass via photocatalysis is an area of expanding research with advances in new technologies and materials with a view toward enhanced sustainability being reported. A significant challenge within this field, however, is understanding the impact photocatalysis has on more recalcitrant compounds present in biomass, such as lignin. Moreover, the current state of lignin model compound research is still largely focused on the breakdown of small models containing typically only one linkage. Described herein is the use of TiO_2_-mediated photocatalysis for the degradation of a representative hexameric lignin model compound which contains multiple linkages (e.g., 5-5′, β-5, and β-O-4). The results revealed that while cleavage of the β-5 and β-O-4 occurred, the 5-5′ appeared to remain intact within the identified reaction intermediates. To understand some of the more fundamental questions, a dimeric compound with a biphenyl linkage was synthesized and studied under photocatalytic conditions. The proposal of intermediates and pathways of degradation based on the studies conducted is presented and discussed herein.

## Abstract 2

TiO_2_-mediated photocatalysis employed for the conversion of a complex lignin model compound with the proposal of products and pathways.

## Introduction

The application of photocatalysis as a sustainable conversion technology for bioenergy and bioproducts has grown significantly in recent years. This is evident from both observing the trends being reported in the literature and the number of excellent review articles that have been published.^1,2^ Initial reports often focused on biomass fingerprint compounds such as sugars^3−6^ and cellulose^2,7−9^ for the generation of H_2_ and/or value-added compounds. Subsequently, this provided the platform for more substantial developments including novel material synthesis,^3^ pretreatment processes,^10−12^ solar activation,^7,13^ and the valorization of raw biomass feedstocks such as woody materials,^14^ grass,^15^ and rice husks.^16^ While these reports represent the advancement of the technology, they also highlighted key challenges which must be addressed, especially in relation to the chemical composition of lignocellulosic materials. The development of photocatalysis as a technique for sustainable biomass valorization is underpinned by not just overcoming these challenges, but also identifying the fundamental chemical processes which influence them. A key example of this is the photocatalytic reforming of lignin, which has rarely been reported in the literature with examples primarily centered on the carbohydrate portion of biomass.^17^ Lu et al. synthesized a composite species consisting of TiO_2_ modified with lignin-based carbon which resulted in the formation of vanillin as the primary product (∼1% yield). In addition, TiO_2_ and H_2_O_2_ were employed for the degradation of rice husk which generated a range of products, with the most abundant being alkanes, phthalates, and ketones.^18^ The pretreatment and subsequent photocatalytic oxidation of commercial lignin liquor have also been reported using ZnO–TiO_2_ nanoparticles, with the authors observing the formation of four main organic acids: ferulic acid, benzoic acid, *p*-coumaric acid, and vanillic acid with reported yields in a range of 3.8–9.71 mg L^–1^.^11^ While these examples demonstrate the feasibility of the process, the low yields reported suggest that photocatalysis was incapable of cleaving all the linkages present within lignin. The bonding patterns and associated bond enthalpies within lignin give rise to the structure being considered as the relatively unreactive portion of biomass. This has been well documented in the literature^19^ with density functional theory calculations demonstrating the strong interunit linkages found within lignin being the reason for its recalcitrance.^20^ In addition, work investigating the cleavage of C–C bonds has also been reported using both kraft lignin^21^ and model compounds.^22^ The study by Shuai et al.^21^ showed that there was a relatively low amount of monoaromatic species produced in the cobalt sulfide-catalyzed degradation of kraft lignin with yields ranging from 1.2 to 13.6%. This study, along with others,^23,24^ underpins the assertion that lignin is an unreactive polymer and that the successful targeted degradation of all of the bonds within it is still a significant and challenging area of ongoing research. Therefore, to develop a (photo)catalytic system which can efficiently cleave all the linkages present within lignin, model compounds have been synthesized^25−29^ which provide an opportunity to study catalytic conversions of lignin functionalities at a fundamental and less complex level. Typically, the synthesis of lignin model compounds has focused on simple dimeric models which contain the β-O-4 linkage^26,30−33^ as it is the most common bonding pattern found within lignin.^34^ The presence of the relatively unstable β C–O ether bond however means that these models are readily degraded under very mild conditions and are therefore not representative of the challenges associated with the native material, particularly when the C–C linkages such as the 5-5′ are considered. In our recent work,^35^ we demonstrated the photocatalytic degradation of the β-5 linkage, which, unlike certain β-O-4 linkages, was stable under UV irradiation. Complete conversion of a model dimer containing the β-5 linkage was achieved within 45 min of irradiation with a removal rate of 6.3 × 10^–3^ mg mL^–1^ min^–1^. The consumption of the β-5 substrate also led to the formation of four reaction intermediates which were identified by liquid chromatography–mass spectrometry (LC–MS) and nuclear magnetic resonance spectroscopy with the first diol species observed within 2 minutes of irradiation. This work was the first to report the degradation of β-5 via photocatalysis and highlighted the need for photocatalytic reactive oxygen species (ROS) (e.g., OH^·^ and O_2_^·^^–^) in lignin degradation. To further advance this work, however, more representative lignin model compounds must be examined. The synthesis of larger, more complex models has only been demonstrated a handful of times^31,36−38^ including work reported by this research group. This work^38^ demonstrated the use of readily scalable chemistry for the synthesis of hexameric and octameric lignin model compounds which themselves contain many of the linkages found within lignin and thus are the most representative set of model compounds in the literature. To date, however, there are no examples which have reported or explored the degradation of such compounds. Therefore, presented in this work, for the first time, is the photocatalytic degradation of a hexameric lignin model which contains the β-O-4, β-5, and 5-5′ biphenyl linkages. Under low-power ultraviolet-light-emitting diode (UV-LED) irradiation and in the presence of TiO_2_, we demonstrate the role photocatalysis plays in the conversion process to reveal a range of reaction intermediates. Despite the complexity of the system and challenging oxidation pathway, the extent of photocatalytic bond cleavage is also explored by closely monitoring the presence of the 5-5′ biphenyl linkage.

## Results and Discussion

Photocatalysis, when applied to lignin model degradation, has the potential to generate several products, many of which have competing and synergistic pathways because of the nonselective nature of the process. Moreover, this point becomes further prevalent when considering a lignin model composed of multiple different linkages. To the best of our knowledge, this has never previously been considered within photocatalytic research as compounds containing only one linkage have been reported. This highlights the novelty of the work reported here for both lignin model photocatalysis and more broadly biomass valorization.

The oligomeric lignin model (referred to hereafter as the *hexamer*) which has been previously synthesized within our group^38^ has a hexameric structure and contains three of the most common linkages found within lignin: β-O-4, β-5, and the 5-5′. The high level of complexity found within the hexamer means that the determination of products arising from the photocatalytic degradation is challenging. In terms of the hexamer, the center of the molecule contains the 5-5’ C–C linkage and is flanked at either side by β-O-4 linkages followed by a β-5 linkage at each end. It should be noted that the β-5 linkages in the hexamer differ slightly from the dimeric model used in the previous study^35^ because of the presence of an alkene in the propyl sidechain in the previous study but not in the hexamer. The presence of the 5-5′ linkage at the center gives rise to axial chirality along and symmetry across this biphenyl bond. The result is that per molecule of hexamer there are two β-O-4 and two β-5 linkages. Although the hexamer can be considered a polyol, there are insufficient OH groups to aid the complete dissolution in 100% H_2_O. Therefore, similar to the β-5 study, the medium used in these photocatalytic experiments was 50% aqueous MeCN.

The synthesis of the hexamer begins with acetovanillone and oxidative dimerization by the action of sodium persulfate and iron sulfate heptahydrate to provide the biphenyl species diapocynin.^39,40^ Following this, the extension of the ketone chain and then bromination provided the activated species, and displacement of the bromide by the phenolic β-5 dimer provided the hexameric skeleton. Subsequent reduction and hydrogenolysis provided the final hexameric species.

**Figure 1:**
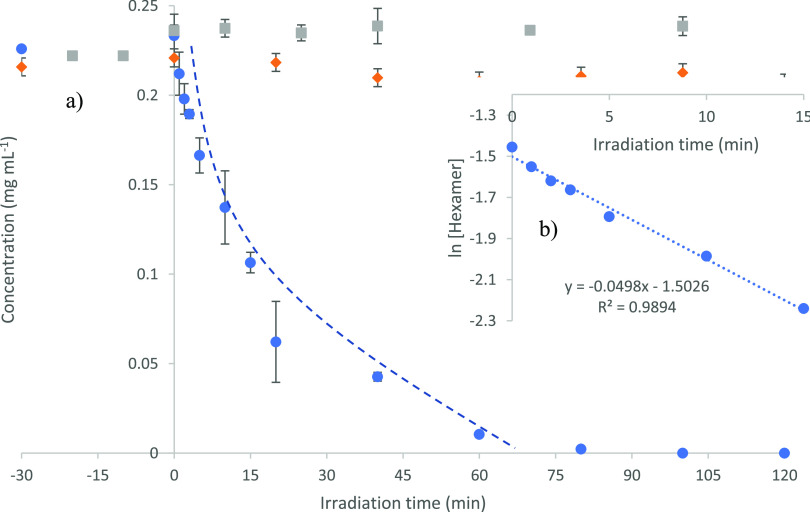
Photocatalytic degradation of the hexameric lignin model compound where (a) is the time-concentration profile under photocatalytic (blue), photolytic (orange), and catalyst only conditions (gray) and (b) is a plot of ln[hexamer] vs time.

Photocatalytic degradation of the hexamer (see the ESI for a full experimental procedure) was achieved (to the limit of detection) within ∼100 min of UV-LED irradiation, Figure 1. Furthermore, photocatalysis was confirmed as the primary mechanism occurring via monitoring the reaction under photolytic (UV only) and dark (catalyst only) control conditions, with both showing no significant removal. The photoactivity of the TiO_2_ catalyst was also monitored over three experimental cycles to confirm that no significant reduction in activity was detected (see the ESI and Figure S4). A plot of ln[hexamer] versus initial irradiation time (Figure 1b) also confirmed a pseudo-first-order reaction with an initial rate constant of 0.0489 min^–1^. In comparison to the previous work, on the β-5 linkage there are differences worth noting. The rate of degradation for the β-5 model was significantly faster (0.0693 min^–1^) than that of the hexamer which was likely due to the increased size and chemical complexity of the latter. Furthermore, while ∼15% adsorption of the β-5 onto the TiO_2_ at equilibrium occurred under dark conditions, no equivalent adsorption was detected for the hexamer.

**Figure 2:**
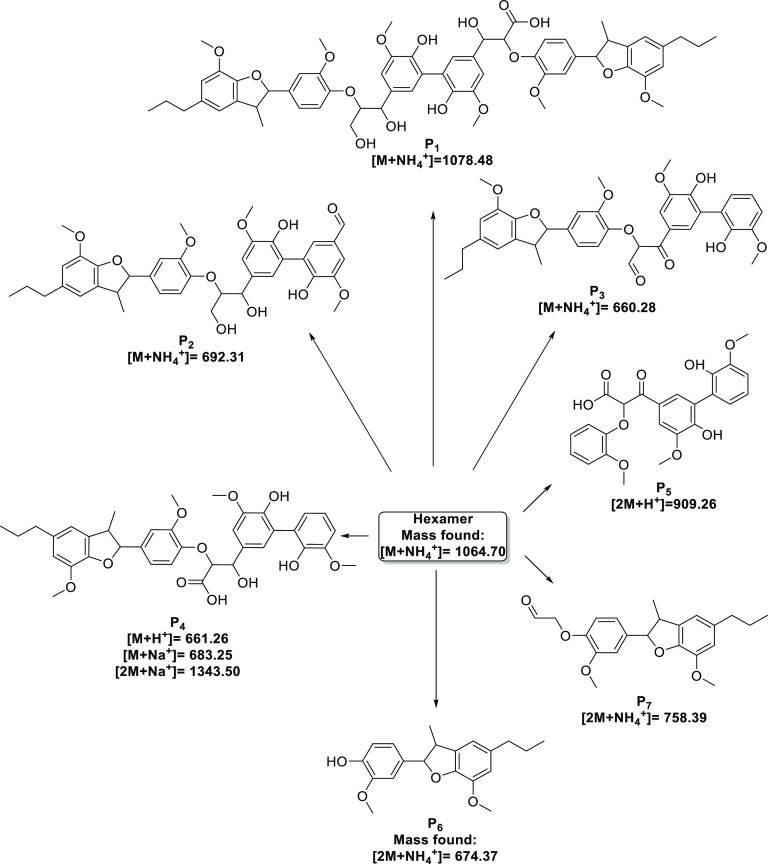
Proposed products arising from the degradation of the hexameric lignin model compound.

This suggests that the mechanism of reaction was via ROS diffusion and attack in the bulk medium as opposed to direct-hole oxidation occurring at the TiO_2_ surface. In relation to reaction intermediates and product generation, oxidation of the hexamer resulted in a range of compounds. Several products from the hexamer were observed by high-performance liquid chromatography (HPLC) analysis with supplementary supporting analysis from LC–MS. A total of seven reaction intermediates (**P_1_**–**P_7_**) were identified with their proposed structures summarized in Figure 2. Because of the complexity of the hexameric structure, fully elucidating the possible pathways is challenging and is therefore part of ongoing work. Presented here are the initial key findings which provide a crucial insight into the fundamental processes which may be occurring. The structures shown in Figure 2 can be rationalized from the known reactivity patterns of the hexamer along with the principles of photocatalysis and are supported by the masses found by LC–MS. The formation of the first proposed intermediate **P_1_** results from the full oxidation of one of the primary alcohol groups in the β-O-4 sidechain in the hexamer to provide the carboxylic acid in the sidechain. Similar photocatalytic oxidation of primary alcohols to provide the corresponding carboxylic acid has previously been reported^41^ with the proposed mechanism of oxidation occurring via the hydroxyl radicals in solution. The next proposed product **P_2_** appears to be the result of C–C bond scission between the α and β carbon in one of the β-O-4 substructures providing the formyl group on the aromatic ring. In addition, **P_7_** could be the complementary fragment arising from the cleavage of this C–C bond. Cleavage of the CH_2_C(O)H side chain of **P_7_** would then lead to the formation of **P_6_**. Although its formation could also be explained by direct cleavage of the β-O-4 C–O bond in the hexamer or indeed from the proposed products **P_1_**, **P_2_**, **P_3,_** and **P_4_**, a plot of the formation of **P_6_** can be seen in the ESI.

It has been reported previously that the oxidation of the α-OH in the β-O-4 linkage can result in fragmentation of the β-C–O bond as is seen in the formation of **P_6_**.^42^ The proposed intermediates **P_3_** and **P_4_** all have the same skeletal structure with the only differences being the level of oxidation at the α- and γ-carbons of the side chains with carboxylic acid formation observed in the case of **P_4_**. **P_4_** could be produced by C–C cleavage between the aromatic ring and the α-carbon of the side chain and also the oxidation of the primary alcohol. The formation of the carboxylic acid species **P_5_** appears to be the result of the initial fragmentation between the α-carbon and the aromatic ring in the β-5 region of the hexamer to give a radical on the aromatic ring; further reaction at the β-O-4 linkage in the molecule to provide the acid would explain the formation of this species.

**Figure 3:**
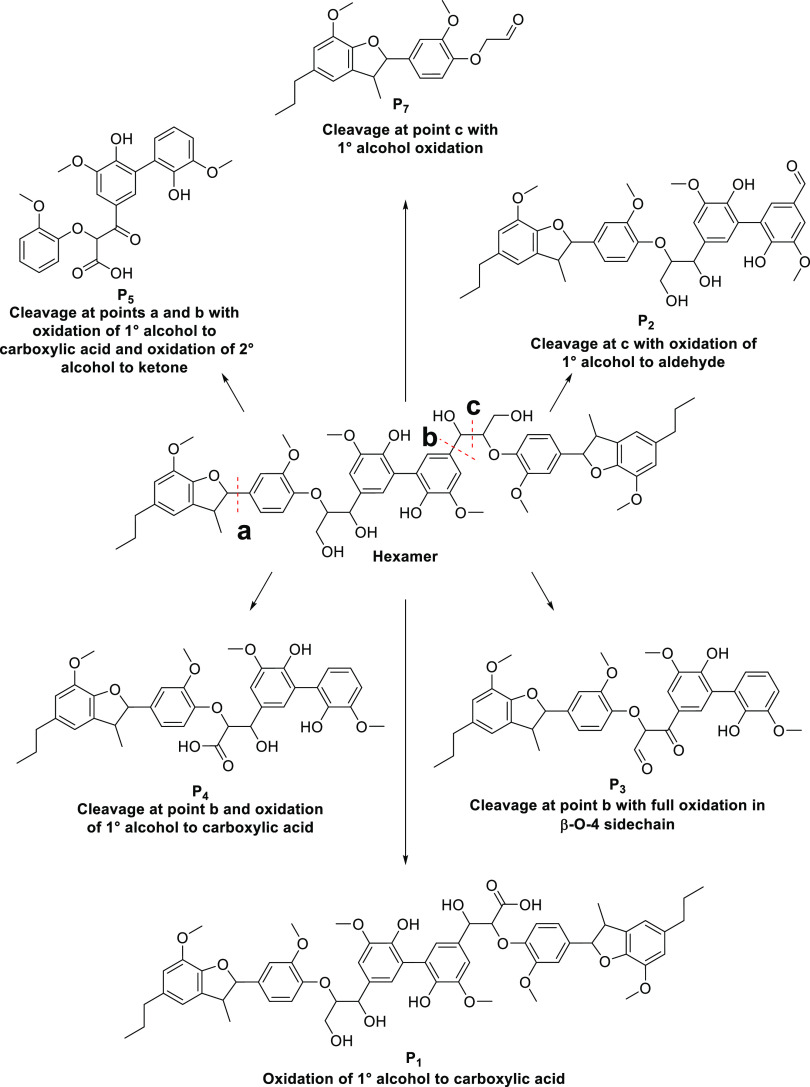
Proposed pathways from the hexamer leading to the formation of intermediates **P_1_**–**P_7_**.

**Figure 4:**
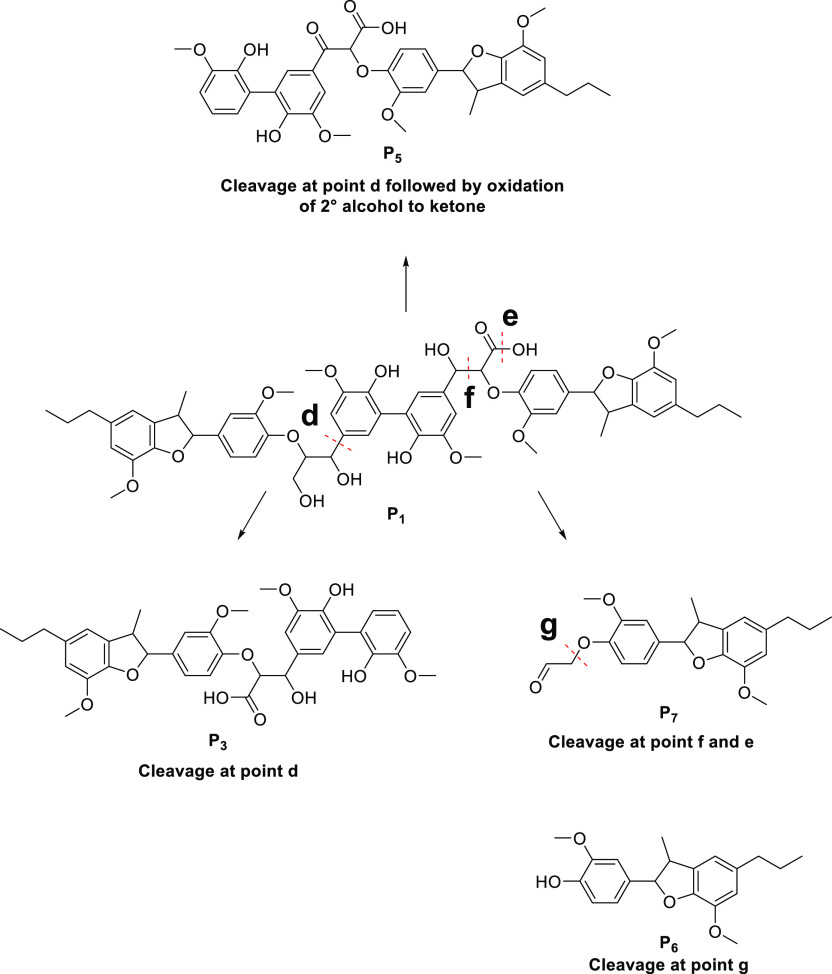
Proposed pathways from **P_1_** leading to the formation of intermediates **P_3_**, **P_5_**, **P_6_**, and **P_7_**.

In terms of rational pathways which can explain the formation of these proposed intermediates, it is envisioned that there are two possible main routes: one beginning with the hexamer and the other beginning with **P_1_**, in which all of the side chain alcohols have been oxidized. Figures 3 and 4 show both the proposed routes.

**Scheme 1:**
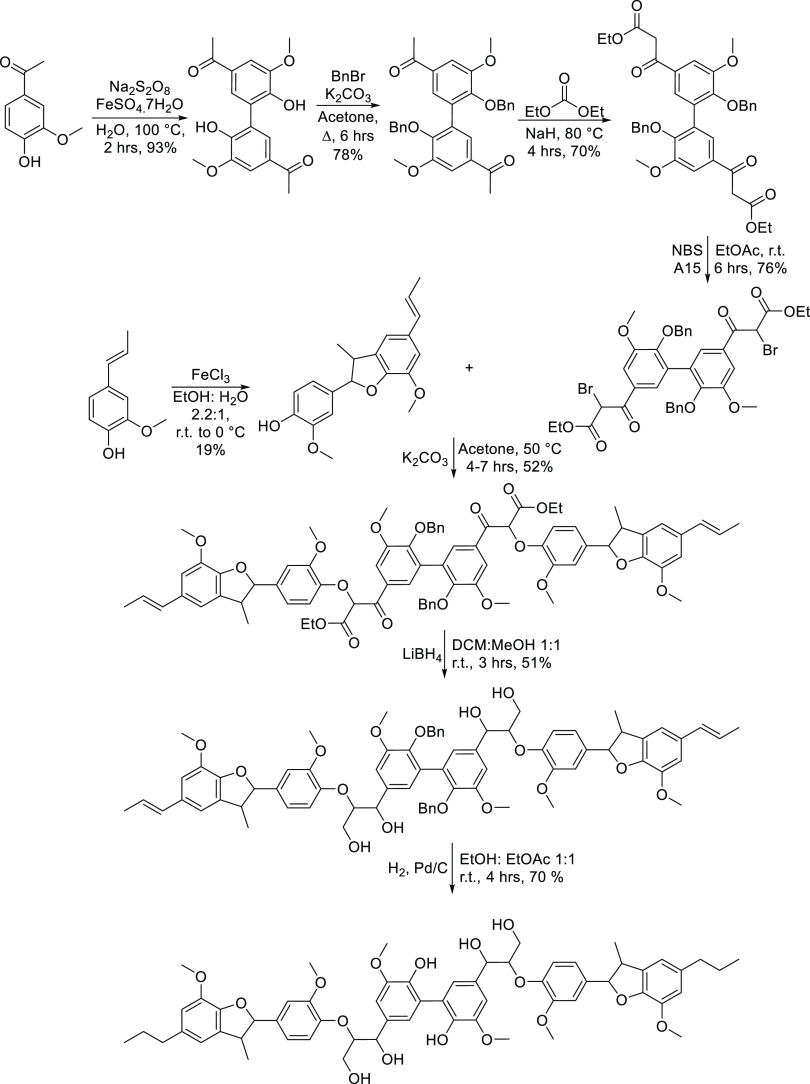
Synthetic Route to the Hexameric Lignin Model Compound

The pathways shown in Figure 3 could account for products **P_1_** to **P_7_** from the hexamer, and the pathways in Figure 4 show potential conversions of **P_1_** to product **P_3_** and then the subsequent cascade of **P_7_** to **P_6_**. There is some potential overlap between the two proposed pathways shown in Figures 3 and 4; for example, **P_3_** and **P_7_** could potentially be produced by either pathway. In the case of **P_6_**, it could be argued that there are several precursors that could lead to its formation, directly from the hexamer via cleavage of the β-C–O bond^43^ but also from **P_1_, P_2_**, **P_3,_** and **P_4_**. As we have previously shown in the β-5 study,^35^ cleavage of the guaiacol- furan C–C bond under photocatalytic conditions can occur, and therefore, the same linkage in this study is presumed to undergo a similar pathway; however, no smaller fragments were detected in the reaction solution. The relative complexity found throughout all of the proposed intermediates is a result of the complexity of the hexameric substrate and in turns helps to demonstrate the feasibility of photocatalysis for the cleavage of particular bonds present within lignin. The β-O-4 sidechain is the center of highest disruption by the photocatalytic process which is in good agreement with many previous studies showing the β-O-4 linkage to be susceptible to cleavage in both models and native lignin.^44−47^ Intermediate **P_5_** is proposed to be the result of the modification to the β-5 linkage present, with cleavage being proposed in the central C–C linkage between the furan and aromatic rings. Deeper elucidation of the mechanism is ongoing, but monitoring is difficult in such a highly complex system. There is a high level of confidence in the formation of the intermediate **P_6_** during the reaction because this compound is also an intermediate in the synthesis of hexamer. The plot of this formation during the 120 min of reaction is shown in the ESI (Figure S3). A key observation, however, is the presence of the 5-5′ linkage in all of the proposed product species, clearly suggesting that it is not susceptible to cleavage via photocatalysis. The prevalence of the 5-5′ linkage has been reported to be ∼5–7% for softwoods and <1% for hardwoods and has a calculated bond enthalpy much greater than the other linkages, ranging from 115 to 118 KJ mol^–1^ depending on the substituents on the ring.^20,48^ While this may present an issue in relation to developing conversion technologies capable of cleaving all linkages present within lignin, it also provides an insight into one of the reasons, aside from the repolymerization aspect of lignin valorization, for the relative unreactive nature of lignin as a whole. To date, there has only been one previous study which investigated the degradation of a model compound containing a 5-5′ linkage using a catalytic system. Machado et al.^43^ monitored the reactivity of their model compound via fluorescence spectroscopy but did not identify the degradation products. Therefore, to validate some of the key findings presented in the proposed reaction schemes detailed in this manuscript, the photocatalytic degradation of a model containing the 5-5′ linkage was also monitored. To determine if this 5-5′ bond is the most recalcitrant within the hexamer, a model was synthesized with inherent functionalities found within the larger biopolymer structure. The initial steps implemented here are the same as those seen previously in Scheme 1, namely, the oxidative dimerization and subsequent benzylation. Ketone reduction and de-O-benzylation provided the dimeric phenolic species in an overall yield of 40.7%. This 5-5′ model compound was treated under the photocatalytic conditions in 50% aqueous MeCN. As initially proposed, the presence of the 5-5′ linkage resulted in less sensitivity toward the photocatalytic conditions.

**Figure 5:**
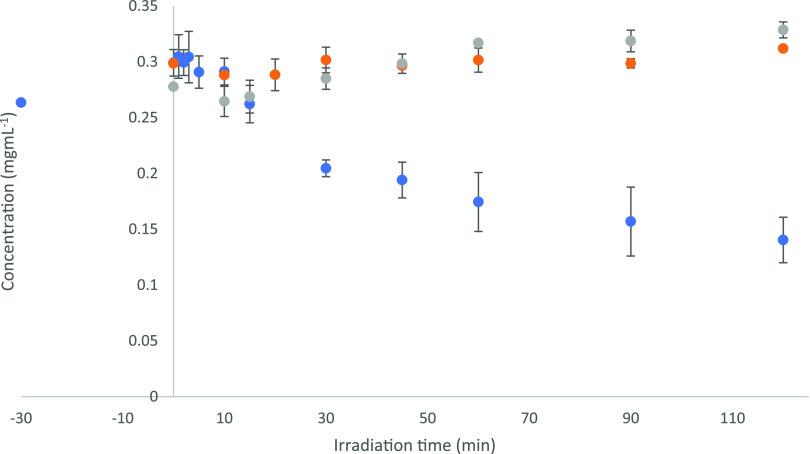
Photocatalytic degradation of 5-5′ biphenyl lignin model compound where (a) is the time-concentration profile under photocatalytic (blue), photolytic (gray), and catalyst only conditions (red).

**Figure 6:**
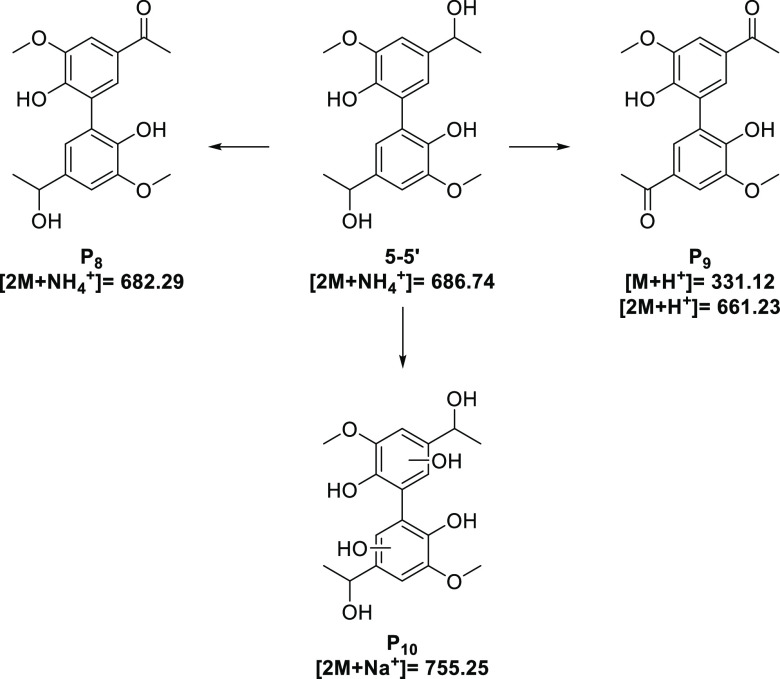
Proposed products **P_8_**–**P_10_** arising from the partial degradation of the 5-5′ model dimer.

Figure 5 shows that, in the presence of UV-LED irradiation and a TiO_2_ catalyst, consumption of the 5-5′ model compound was not complete after 120 min. No reaction was observed under photolytic (UV only) and/or dark (catalyst only) control conditions, again suggesting that photocatalysis was the primary mechanism. An initial rate of 3.23 × 10^–3^ mg mL^–1^ min^–1^ showed a reduced rate of reaction compared with that observed for the hexamer degradation or for the β-5 substrate in the previous study. An overall decrease in the concentration of the 5-5′ model compound of 0.162 mg mL^–1^ (45.5% reduction) during the 120 min of irradiation implies that a level of resistance was conferred to the substrate, potentially as a result of the strong C–C linkage between the phenyl rings. To determine if any cleavage of the biphenyl bond was occurring, the reaction progress and formation of products were monitored by HPLC and LC–MS. Results of LC–MS analysis (Figure 6) suggested two major products based on detected masses and also a difference in polarity. The oxidation of the secondary alcohols in one or both sidechains of the 5-5′ model can be seen in both **P_8_** and **P_9_** which is in good agreement with the formation of **P_1_**, **P_2_**, **P_3,_** and **P_4_** from the hexamer.

**Scheme 2:**
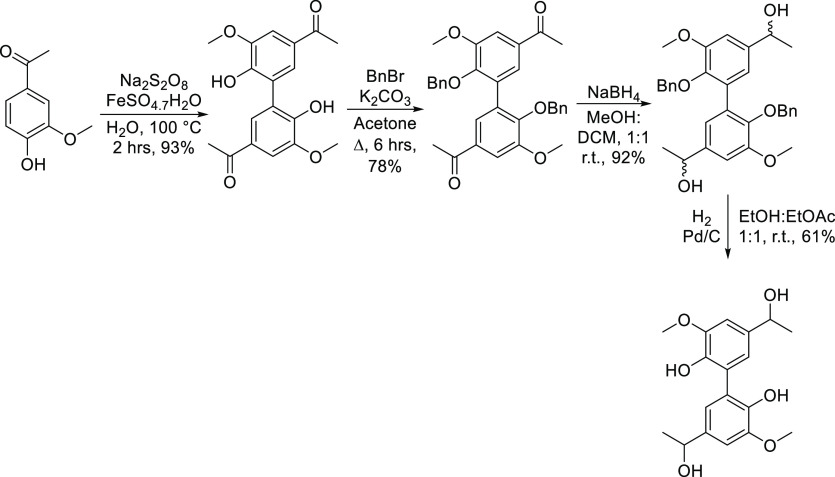
Synthetic Route Implemented for the Synthesis of the 5-5′ Biphenyl Model Dimer

The other proposed product formed during the reaction is in agreement with the addition of two hydroxyl groups to the substrate. It is challenging to determine the positions on the substrate where the hydroxyl groups have been added, but there is literature precedent for both ring hydroxylation and also sidechain oxidation in the presence of hydroxyl radicals.^49,50^ The formation of the three proposed product species, **P_8_**–**P_10,_** helps to develop the idea that throughout the reaction course there is no formation of monomeric phenolic species from the biphenyl core by cleavage of the 5-5′ linkage. The use of this biphenyl model compound has shown that the presence of a strong C–C linkage like the 5-5′ can prevent complete destruction of lignin models under the photocatalytic conditions (Scheme 2). Moreover, this also suggests that it has the potential to be the only remaining lignin-type linkage from larger species This potential limitation not only has implications in this study but also to the application of the technology for deployment with native lignin and/or even biomass as a whole.

The literature has demonstrated that photocatalysis can cleave the linkages present within cellulose and hemicellulose.^51^ The linkages present in lignin, however, prove to be more challenging because of the random nature of its composition. This is a crucial insight with regard to the feasibility of deploying photocatalytic technology for biomass valorization. The data showed here suggest that photocatalysis may be incapable of complete lignin conversion which subsequently could influence the yield of desirable product streams, for example, hydrogen generation. Although the work presented in this hexamer study may have highlighted a potential limitation to the technology, an example of such a complex model of lignin in a photocatalytic system has never been explored before. Therefore, this investigation has shown that the more representative the model is of native lignin, the less accessible all of the linkages are to being cleaved. Furthermore, monitoring the mechanism could facilitate the future design of a system which is capable of selectively cleaving linkages present within the lignin structure. Currently, within the field of lignin catalysis as a whole, the selectivity of the linkages is low, with the weaker bonds, that is, β-O-4 being cleaved first and the stronger being left behind. This study gives an insight into the linkages that remain and subsequently what occurs when these linkages are exposed to a photocatalytic system.

## Conclusions

We have reported for the first time the use of TiO_2_-mediated photocatalysis for the degradation of a hexameric lignin model compound containing multiple functionalities. The resulting proposed intermediate and product species have been rationalized by employing LC–MS and known photocatalytic transformations. Significant degradation and a tandem reduction–oxidation reaction on the sidechains of the hexamer is proposed in this work. The calculated bond enthalpy of the 5-5′ biphenyl linkage is the proposed reason for the photocatalysis being unable to break the key C–C linkage, supported by exposing a simple 5-5′ model to the reaction conditions which indicated that the biphenyl linkage is left intact following 120 min of irradiation. These findings provide a valuable and fundamental insight into the feasibility of photocatalytic technology for both the conversion of lignin and the sustainable valorization of biomass.

## Supporting Information Available

An image of the reactor under irradiation and the spectral output of the UV-LED array; calibration curves for (a) hexamer, (b) 5-5′, and (c) **P_6_**; structures and associated mass spectra of intermediates **P_1_–P_10_**; generation of reaction intermediate **P_6_**; activity of the TiO_2_ photocatalyst over 3 reaction cycles (PDF)
