# Supplementary material for: Toward the Photocatalytic Valorization of Lignin: Conversion of a Model Lignin Hexamer with Multiple Functionalities
Source: ACS Sustain Chem Eng. 2022 Sep 2;10(37):12107–16. doi: 10.1021/acssuschemeng.2c01606 (PMC9490757; doi:10.1021/acssuschemeng.2c01606)
Supplement: Supplementary file 1 — sc2c01606_si_001.pdf [file sc2c01606_si_001.pdf]

Supporting Information

Towards the Photocatalytic Valorisation of Lignin: Conversion of a Model Lignin Hexamer with Multiple Functionalities

Christopher W. J. Murnaghan*, Nathan Skillen*, Bronagh Hackett*, Jack Lafferty*, Peter K. J. Robertson* and Gary N. Sheldrake*,†

**School of Chemistry and Chemical Engineering, Queen's University Belfast, David Keir Building, Stranmillis Road, Belfast, BT9 5AG, UK.,*

†Corresponding author: Gary N. Sheldrake – *School of Chemistry and Chemical Engineering,*

Queen's University Belfast, David Keir Building, Stranmillis Road Belfast, BT9 5AG, UK.

g.sheldrake@qub.ac.uk

Total number of pages : 22

Total number of figures : 14

Contents

Figure S1: (a) an image of the reactor under irradiation and (b) the spectral output of the UV-LED array indicating a peak at ~370 nm 4

Figure S2: Calibration curves for (a) hexamer, (b) 5-5' and (c) a b-5 model dimer (identified as P6) 5

Figure S3. Structure and associated mass spectrum of intermediate **P_1**..12

Figure S4. Structure and associated mass spectrum of intermediate **P_2**..13

Figure S5. Structure and associated mass spectrum of intermediate **P_3**..14

Figure S6. Structure and associated mass spectrum of intermediate **P_4**..15

Figure S7. Structure and associated mass spectrum of intermediate **P_5**..16

Figure S8. Structure and associated mass spectrum of intermediate **P_6**..17

Figure S9. Structure and associated mass spectrum of intermediate **P_7**..18

Figure S10. Structure and associated mass spectrum of intermediate **P_8**......................................19

Figure S11. Structure and associated mass spectrum of intermediate **P_9**......................................20

Figure S12. Structure and associated mass spectrum of intermediate **P_{10}**....................................21

Figure S13: Generation of reaction intermediate P6 (b-5 model dimer) during the photocatalytic conversion of the hexameric substrate 22

Figure S14: Activity of the TiO_2 photocatalyst over 3 reaction cycles. Fresh hexamer substrate and reaction solvent were added between each cycle. Trendlines are added into each cycle for clarity. 22

Materials and Methods

For synthetic work performed, all reagents were used without further purification and bought fresh for this study. All reagents and all solvents were bought from either Sigma-Aldrich or Fisher Scientific. Ethanol used in reactions was absolute ethanol and was bought from Fischer scientific. SiO_2 plug and column chromatography was performed using Fluorochem silica gel 60A. Water used was distilled water and stock solutions made up by dissolving substrate in organic phase and water added and allowed to equilibrate for 3 hours prior to use in a sealed bottle.

Analysis

NMR spectroscopy was performed on Bruker Avance 400 MHz with $CDCl_3$ and CD_3CN as the deuterated solvent and stated in the text where these were used. The standard used was tetramethylsilane and set at 0 ppm, $CDCl_3$ was set at 7.26 ppm and CD_3CN was set at 1.93 ppm.

For HPLC–UV analysis, an Agilent 1100 HPLC system equipped with a photodiode array UV–Vis was used. A Phenomenex KinetexR C18 analytical column (150 mm × 4.6 mm, 100 Å) was used, and the mobile phase was (A) 0.2% acetic acid and (B) methanol over the following gradient: 50:50 (A: B) to 100% B (0–6 min) and then back to 50:50 (A: B) (6–10 min). The flow rate was 1 mL min^{-1}, and the injection volume was 5 µl.

LC–MS analysis was performed on an Agilent 1260 Infinity II with an InfinityLab LC/MSD equipped with a Kinetex 5 µm C18, LC column 150 × 4.6 mm. Chromatography was carried out using two solvents: (A) water and 0.1% formic acid solution and (B) acetonitrile and 0.1% formic acid solution. The gradient followed is 0–6 min 50% solvent A 50% solvent B and 6–12 min 100% solvent B. The flow rate was 1.0 mL min^{-1} with an injection volume of 5 µl, and the column temperature was 25 ◦C. The detector on the LC was a VWD measuring the wavelength at 275 nm. The mass detector was in negative mode and the machine is ESI-MS. Preparative-HPLC separations (which were required for the final preparation of the hexamer) were preformed using an Agilent 1290 Infinity II preparative-HPLC system, equipped with a Phenomenex Kinetex 5 µm Phenyl-Hexyl column (250 x 21.2 mm). The chromatographic conditions used for gradient elution were as follows: flowrate 25 mL/min; injection volume 500 µl; UV detection 250 nm. The gradient started at 65 % (v/v) acetonitrile and was maintained isocratically for the first minute, thereafter the acetonitrile content was raised linearly to 100 % within 10 minutes. Finally isocratic elution of 100 % acetonitrile was maintained for a further three minutes, with a total run time of 13 minutes.

Photocatalysis Experiments and Procedure

Photocatalytic experiments were carried out in a small-scale experimental set up that consisted of a bespoke stirred borosilicate glass reactor (see Figure S1 (a)). In a typical experiment, a predetermined weight of lignin model substrate was dissolved in a 30 mL solution of 50:50 water and CH_3CN to equal a final concentration of 0.25 mg mL^{-1}. In addition, a loading of 0.5 g L^{-1} TiO_2 P25/P20 (Evonik) was also added to the reaction solution. The reaction solution was continuously stirred throughout the experiment using a magnetic stirrer bar and plate. Each reaction was kept in the dark for 30 min prior to irradiation to allow for equilibrium. The length of time required to reach equilibrium was determined by conducting a '*dark*' control experiment (i.e., in the absence of light). Irradiation was

provided by a novel spiral UV-LED array (Lighting Will), which provided 360 ° irradiation of the reactor. The irradiated surface area of the reactor was determined to be 70.65 cm^2. The LEDs were mounted onto a stainless-steel mesh support and had a peak wavelength in the range of 365-370 nm (see Figure S1 (b)), which was determined using a StellarNet Black Comet spectrometer. The LED array was operated at V_F =12.0 dcV and I_F= 0.4 A which gave an overall electrical power of 4.8 W. The irradiance at the reactor was 3.8 mW/cm^2 and was measured using a UV radiometer (Model UVX by UVP). Samples (1.5 mL) were taken periodically during irradiation and centrifuged at 15 000 rpm for 15 min to separate the supernatant from the catalyst prior to analysis. All experiments were conducted in duplicate (n = 2) and control experiments were performed in the absence of light (dark control) and catalyst (photolysis/light control). The concentration of the hexamer, 5-5' and β-5 model dimer (*i.e.,* P6) were determined using calibration graphs of standards with known concentrations (see Figure S2). In addition, to monitor the activity of the photocatalyst a prolonged reaction was conducted over 3 experimental cycles with each cycle consisting of a 30 min dark equilibrium time followed by 1 hr UV-LED irradiation. At the end of each cycle, the entire reaction solution was removed and centrifuged for 5 mins at 25 000 rpm, with the supernatant removed and prepared for analysis. The remaining catalyst was then resuspended in fresh solvent containing the hexameric substrate before beginning the next cycle.

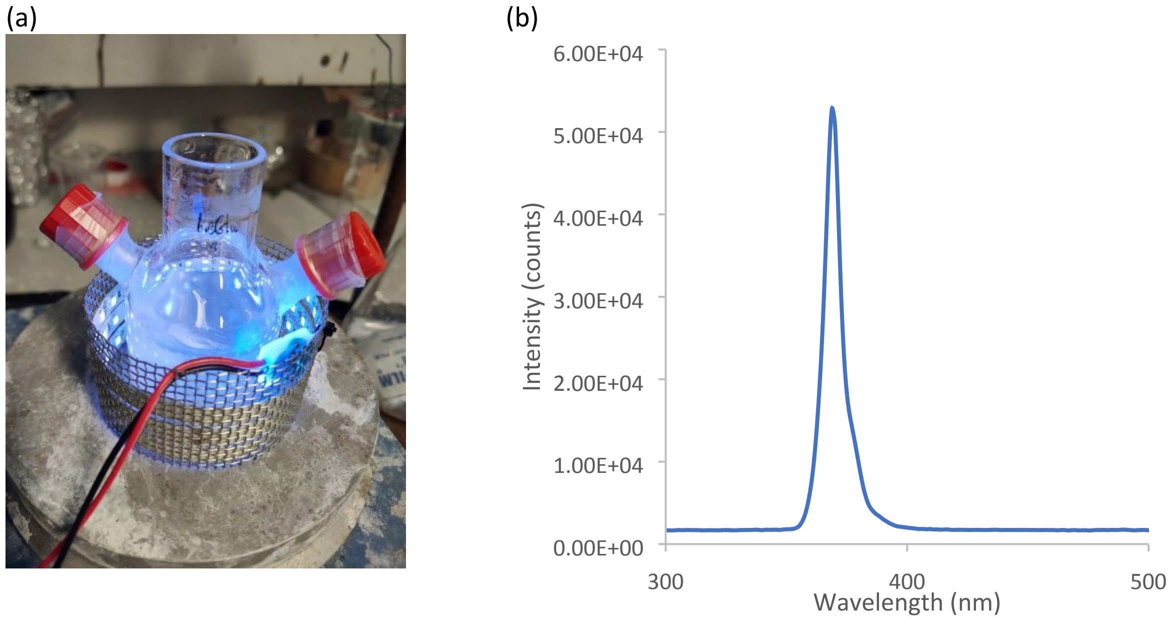

Figure S1: (a) an image of the reactor under irradiation and (b) the spectral output of the UV-LED array indicating a peak at ~370 nm

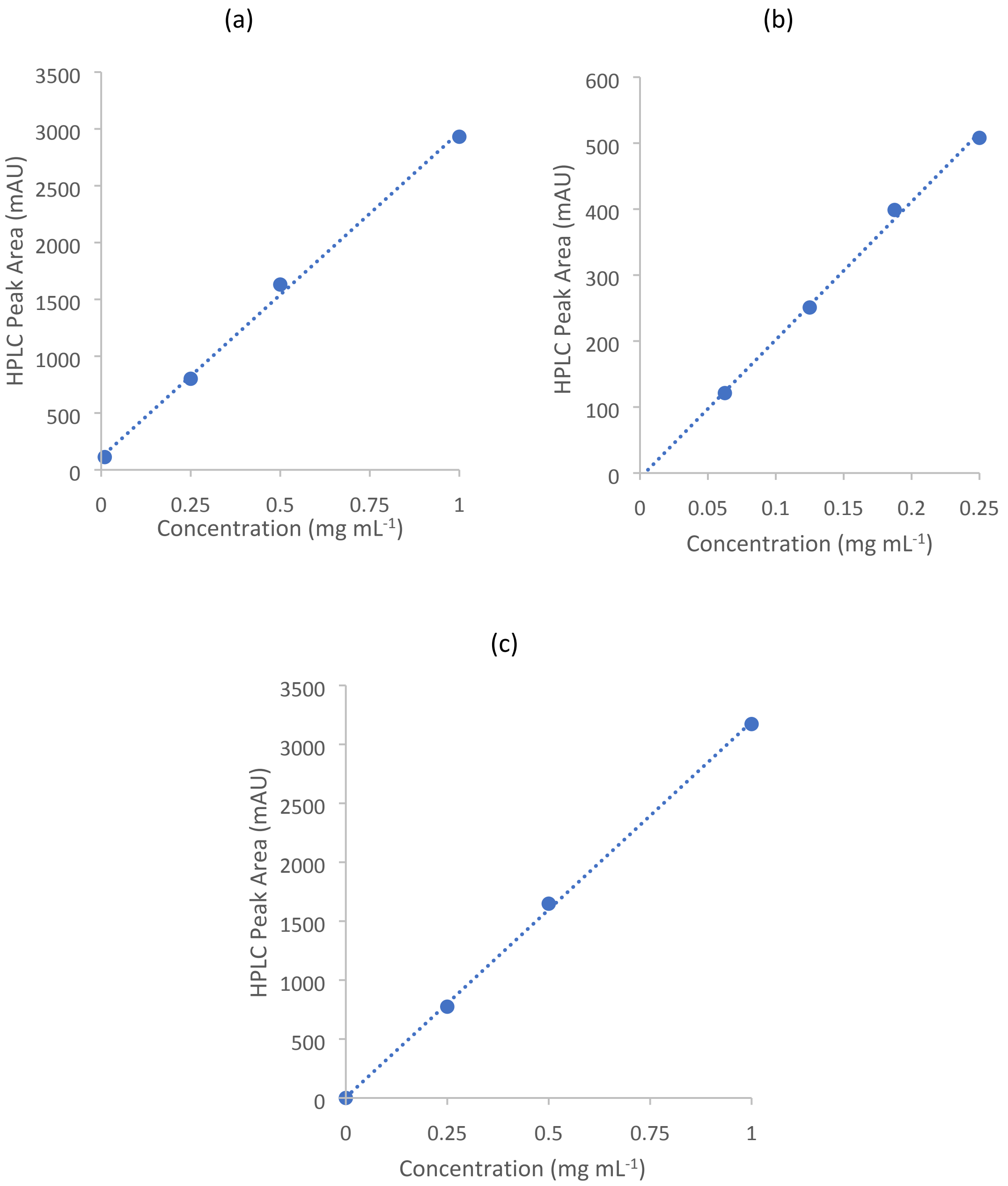

Figure S2: Calibration curves for (a) hexamer, (b) 5-5’ and (c) a b-5 model dimer (identified as P6)

Synthetic schemes and experimental details

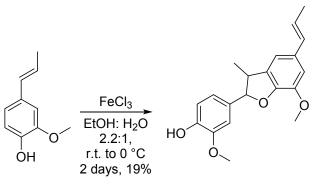

Isoeugenol (5.0 g, 30 mmol) was dissolved in water and EtOH (25 mL and 57 mL, respectively). To this was added FeCl3(2.96 g, 18.2 mmol, 0.6 eq). The flask was stoppered and shaken vigorously for 30 s and then stored in the freezer for 3 d. The mixture was filtered under vacuum and the solid washed with ice-cold EtOH. The crystalline product was then resuspended in a minimum quantity of DCM and passed through a short silica plug, where then the plug was washed with DCM (100 mL) and the filtrate concentrated to provide the product as a white solid (0.94 g, 19% yield).

^{1}H NMR (400 MHz, $CDCl_3$) δ ppm 1.38 (3 H, d, J=6.77 Hz, γ'-H), 1.87 (3 H, d, J=6.53 Hz, γ''-H), 3.45(1H, m, β'-H), 3.88 (3 H, s, OMe), 3.89 (3 H, s, OMe), 5.10 (1H, d, J=9.45Hz, α'-H), 5.62 (1H, s, OH), 6.12 (1H, m, J=15.68 Hz, 6.59 Hz, Ar), 6.90 (2H, s, Ar), 6.97 (1 H, s, Ar).

^{13}C NMR (400 MHz, $CDCl_3$) δ ppm 17.57, 18.40, 45.64, 55.94, 55.99, 93.92, 108.93,109.23, 113.32, 114.08, 120.00, 123.52, 130.94, 132.1, 132.22, 133.28, 144.17, 145.79, 146.59, and 146.68.

Values consistent with reported literature[1,2]

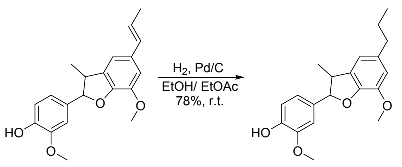

β-5 (1.09 g, 3.34 mmol) added to flask and made solution by addition of EtOH and EtOAc (20 mL each respectively to total 40 mL) and the flask was then purged with nitrogen three times before addition of Pd/C (10% loading, 0.109 g). Following this, the flask was evacuated three times under vacuum and then purged ten times with hydrogen by balloon. The reaction was allowed to stir at room temperature for 3-4 hours before the reaction solution was passed through a pad of celite and the filtrate stripped down to provide a brown/ black oil which was chromatographed on silica gel with elution gradient 7:1 to 5:1 P.E: EtOAc to provide the product as an off-white solid.

^{1}H NMR (400 MHz, $CDCl_3$) δ ppm 0.96 (t, 3H,, J= 7.40 Hz, γ''-CH_3), 1.37 (d, 3H, J= 6.86 Hz, γ'-CH_3), 1.64 (m, 2H, β''-CH_2), 2.55 (t, 2H, J= 7.58 Hz, α''-CH_2), 3.44 (m, 1H, β'-H), 3.88 (os, 6H, 2x OMe), 5.08 (d, 1H, J= 9.53 Hz, α'-H), 5.61 (s, 1H, ArOH), 6.60 (d, 2H, J= 13.61 Hz, Ar), 6.90 (m, 2H, Ar), 7.00 (d, 1H, J= 1.67 Hz, Ar)

^{13}C NMR (300 MHz, $CDCl_3$) δ_H ppm 13.90, 17.40, 25.07, 38.09, 45.80, 55.99, 56.02, 93.62, 108.99, 111.83, 114.04, 115.44, 119.99, 131.94, 132.97, 136.32, 143.87, 145.39, 145.73, 146.66

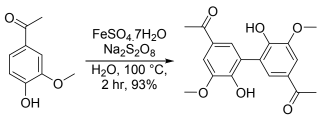

Acetovanillone (10 g, 60.2 mmol) was added to 1 L flask and made into solution by addition of boiling water (300 mL), when complete dissolution was achieved iron sulphate heptahydrate (0.837 g, 3.01 mmol, 0.05 eq) followed immediately by sodium persulphate (7.19 g, 30.2 mmol, 0.5 eq) which turned the solution from deep green to produce a brown precipitate. Precipitate filtered under vacuum and then suspended in boiling water, to boiling water suspension was added solid sodium hydroxide until precipitate dissolved, final precipitation was performed by adding a few drops of concentrated hydrochloric acid where solid was then filtered under reduced pressure and then stored in oven overnight to dry solid of water.

^{1}H NMR (400 MHz, $CDCl_3$) δ ppm 2.58(6H, s, COMe), 4.02(6H, s, OMe), 6.32(2H, s, OH), 7.61(4H, d, J=10.01Hz, Ar)

^{13}C NMR (400 MHz, $CDCl_3$) δ ppm unsuccessful due to insolubility issues in all available deuterated solvents

^{1}H NMR shifts consistent with reported literature [3]

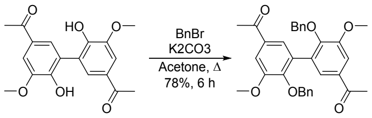

Dedihydroacetovanillone (5g, 15.1 mmol) dissolved in acetone 50mL and brought to reflux, K_2CO_3 (4.17g, 30.2 mmol, 2 eq) added followed by BnBr (3.6 mL, 30.3 mmol, 1 eq) allowed to reflux overnight. Reaction mixture cooled and the filtered, filtrate concentrated to black oil, oil dissolved in EtOAc and passed through large silica plug, filtrate then concentrated to orange oil which crystallised on standing.

^{1}H NMR (300 MHz $CDCl_3$) δ_H ppm 2.47(6H, s, COMe), 3.97(6H, s, OMe), 4.89(2H, s, CH_2Ph), 6.99-7.03(4H, m, Ar), 7.14-7.16(6H, m, Ar), 7.42(2H, s, Ar), 7.61(2H, s, Ar)

^{13}C NMR (300 MHz $CDCl_3$) δ_H ppm 26.24, 55.98, 74.51, 110.72, 125.11, 128.67, 128.94, 131.87, 132.54, 137.06, 149.84, 152.95, 196.81

MS calculated 510.59, mass found [M+ H^+] = 511.21

Values obtained are consistent with reported literature [2]

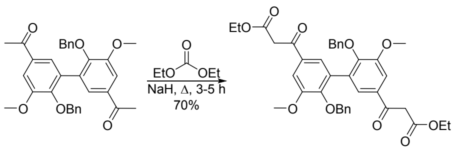

Dibenzylated species (10g, 19.6 mmol) was dissolved in diethyl carbonate (22.85mL, 188.6 mmol, 9.6 eq) and heated to 80°C, to this solution was added sodium hydride (60% dispersion in oil, 3.9g, 97.5 mmol, 4.97 eq) with caution, reaction monitored by NMR spectroscopy. When finished, the reaction mixture was cooled and carefully quenched with water (10mL), then diluted with EtOAc (50mL) and diluted further by the addition of water (40mL). Organic product extracted and washed with water, organic product concentrated to produce dark brown oil, trituration with hexane or petroleum ether

was used to remove excess diethyl carbonate. Flash column chromatography with elution gradient 5:1 to 3:1 P.E: EtOAc, was used to purify further the product, providing white solid.

^{1}H NMR (300 MHz, $CDCl_3$) δ_H ppm 1.26 (6H, t, 7.04 Hz, CH_3), 3.82, (4H, s, CH_2COOEt), 3.98 (6H, s, OMe), 4.18 (4H, q, 7.14 Hz, CH_2CH_3), 4.88 (4H, s, OCH_2Ar), 6.98-7.00 (4H, m, Ar), 7.15-7.18 (6H, m, Ar), 7.29 (2H, s, Ar), 7.61 (2H, s, Ar)

^{13}C NMR (300 MHz, $CDCl_3$) δ_H ppm 14.12, 45.59, 65.13, 61.43, 74.66, 111.23, 125.03, 127.92, 128.1, 128.14, 131.42, 131.94, 136.95, 150.45, 153.14, 167.53, 191.50

MS calculated 654.71, mass found [M+ Na^+] = 677.24

Values obtained are consistent with reported literature [2]

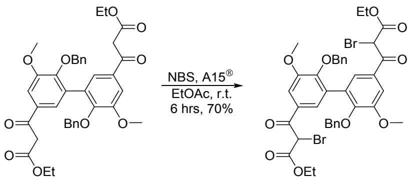

Flask charged with bis-β-keto ester (8.11 g, 12.4 mmol) and made into solution by addition of EtOAc (200 mL), to yellowish solution was then added N-bromosuccinimide (4.34 g, 24.4 mmol, 2 eq) followed by Amberlyst A15 (1.6 g). Yellow solution then allowed to stir at room temperature for 6 hours or until complete consumption of starting material was observed by TLC with eluent 2:1 P.E: EtOAc anisaldehyde stain. When reaction was complete, solid acid catalyst was filtered off and the reaction mixture stripped down to provide orange oil, this was chromatographed on silica gel to provide the product as a yellow oil in 70 % yield.

^{1}H NMR (300 MHz, $CDCl_3$) δ_H ppm 1.23 (6H, t, 7.04 Hz, CH_3), 3.98 (6H, s, OMe), 4.25 (4H, q, 7.14 Hz, CH_2CH_3), 4.91 (4H, s, OCH_2Ar), 5.53 (2H, s, CHBr), 6.99-7.02 (4H, m, Ar), 7.14-7.20 (6H, m, Ar), 7.39-7.40 (2H, s, Ar), 7.64 (2H, s, Ar)

^{13}C NMR (300 MHz, $CDCl_3$) δ_H ppm 13.94, 14.21, 21.06, 45.87, 56.17, 60.41, 63.30, 74.76, 112.31, 125.38, 127.95, 128.08, 128.17, 128.59, 131.89, 136.83, 150.98, 153.19, 165.13, 171.19, 187.12

MS calculated 812.50, mass found [M+H^+] = 830.72

Values obtained are consistent with reported literature [2]

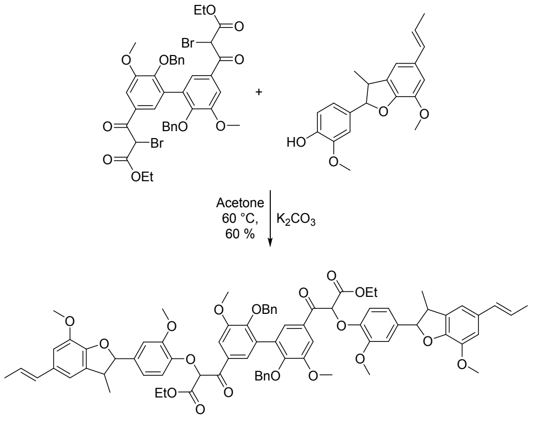

Flask charged with dehydrodiisoeugenol (3.38 g, 0.01 mol) and made into solution by addition of acetone (30 mL), to colourless solution was then added K_2CO_3 (1.38 g, 0.01 mol, 1eq). Brominated intermediate (4.21 g, 0.005 mol, 0.5 eq) dissolved in acetone (20 mL) and added in one portion to dehydrodiisoeugenol solution. Reaction allowed to stir at 60 °C and monitored by TLC with eluent 3:1 P.E: EtOAc anisaldehyde stain. When consumption of dibromo intermediate was observed by TLC the reaction mixture was filtered under gravity and filtrate stripped down to produce dark oil which was chromaographed on silica gel with elution gradient 6:1 to 4:1 P.E: EtOAc to provide the product as an off-white solid in 60% yield.

^{1}H NMR (400 MHz, $CDCl_3$) δH (ppm): 1.17 (6H, t, J = 7.76 Hz, COOCH2CH3), 1.34 (6H, d, J = 7.52 Hz, γ′-H), 1.86 (6H, two doublets superimposed, J= 6.76 Hz γ′′-H), 3.37 (2H, m, β′-H), 3.62-3.94 (multiple singlets corresponding to ArOMe), 4.18 (4H, q, J= 7.00 Hz, COOCH2CH3), 4.88 (4H, overlapping singlets, OCH2Ar), 5.06 (2H, 3 sets of overlapping doublets, J= 9.04 Hz, α′-H), 5.75 (2H, two overlapping singlets, β-H), 6.09 (2H, dq, J= 6.9, 9.00 Hz, β′′-H), 6.35 (2H, d, J= 6.35 Hz, α′′-H), 6.73-7.11 (20H, m, Ar), 7.70 (2H, m, Ar), 7.79 (2H, m, Ar)

^{13}C NMR (100 MHz, $CDCl_3$) δC ppm- 14.16, 17.92, 18.51, 45.75, 45.78, 55.83, 56.07, 56.20, 62.25, 74.71, 82.47, 93.34, 109.40, 110.63, 110.73, 113.16, 113.46, 119.03, 119.14, 123.69, 125.97, 127.83, 127.92, 127.99, 128.03, 128.16, 129.63, 131.04, 132.45, 132.59, 132.62, 133.22, 136.43, 137.30, 144.28, 146.15, 146.62, 150.72, 150.79, 151.13, 152.89, 167.01, 190.32

MS calculated 1303.46, mass found [$M+NH_4^+$] = 1321.73

Values obtained are consistent with reported literature [2]

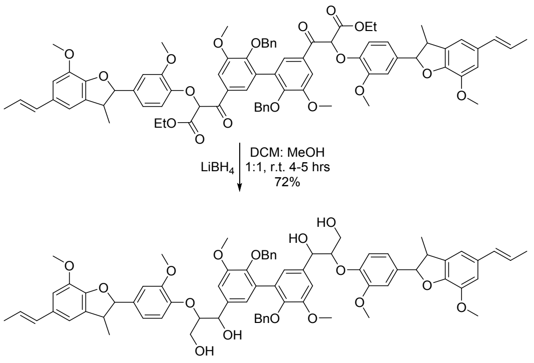

Flask charged with ketoester (0.5 g, 3.84 mmol) and made into solution by addition of methanol (10 mL) and DCM (10 mL), when complete dissolution was observed, the solution was cooled to 0 °C. Lithium borohydride (8.35 mg, 3.84 mmol, 1 eq) was added in one portion and the reaction monitored by TLC with eluent 10:1 DCM: MeOH anisaldehyde stain. When complete consumption of starting material was seen the reaction was quenched by addition of H_2O (15 mL), crude product was extracted with ethyl acetate and washed thoroughly with water and brine (2x 20 mL each). Crude organic dried over $MgSO_4$, filtered and concentrated under reduced pressure to provide the crude as a colourless oil which was chromatographed on silica gel with elution gradient 30:1 DCM: MeOH isocratic solution to provide the product in 72 % yield.

^{1}H NMR (400 MHz, $CDCl_3$) δH ppm 0.88 (d, J= 7.2 Hz, γ′-H-cis)1.38 (6H, two overlapping doublets, J= 6.80 Hz, γ′-H) , 1.86 (6H, two overlapping doublets, J= 6.52 Hz, γ′′-H), 3.43 (m, 2H, J= 7.04 Hz,β′-H) , 3.49-3.79 (region is shown to be overlapping γ-H from a mixture of erythro and threodiastereoisomers as well as containing β′-H-cishydrogen) , 3.82-3.88(multiple singlets corresponding to ArOMe), 3.98 (m, β-H from Threo diastereoisomer), 4.12(broad m, A ring β-H from Erythrodiastereoisomer), 4.76 (4H, overlapping signals, CH2Ar), 4.95 (2H, overlapping doublets, J= 5.10 Hz, α′-H), 5.10 (2H,

overlapping signals, β-H),5.74 (d, J=7.2 Hz, α′-H cis), 6.11(2H, overlapping doublet of quintets, J= 6.52, 15.56 Hz, β′′-H), 6.35 (2H, overlapping doublets, J= 15.56 Hz, α′′-H), 6.75-7.14 (m, Ar)

^{13}C NMR (100 MHz, $CDCl_3$) δC ppm- 17.88, 18.44, 45.71, 45.75, 56.01, 56.05, 56.13, 60.77, 72.90, 72.98, 74.59, 87.03, 93.24, 93.33, 109.37, 109.40, 109.71, 110.22, 110.27, 110.33, 113.42, 119.85, 119.90, 120.49, 121.25, 123.64, 127.53, 127.75, 127.85, 127.94, 128.06, 130.97, 132.44, 132.73, 133.16, 136.24, 137.89, 144.21, 145.13, 146.53, 146.88, 151.57, 151.64, 153.16

MS calculated 1223.42, mass found [M+NH_4^+] = 1241.68

Values obtained are consistent with reported literature [2]

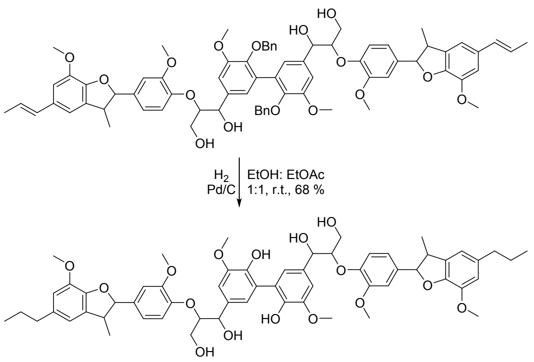

Flask charged with polyol (200 mg, 1.63 mmol) and made into solution by addition of EtOH (10 mL) and EtOAc (10 mL), flask purged with N_2, and 10% Pd/C (20 mg) was added to solution. Flask was then purged with H_2 eight times and allowed to stir at room temperature until complete consumption of the starting material was observed by TLC with elution gradient 5:1 DCM: MeOH anisaldehyde stain. When complete consumption was observed, reaction solution was passed through short celite pad under light vacuum, filtrate stripped down to provide dark oil which was chromatographed on silica gel with elution gradient 25:1 to 20:1 DCM: MeOH to provide the product in 68 % yield.

^{1}H NMR (400 MHz, $CDCl_3$): δ 0.79 (d, J= 7.3 Hz, γ′-H-cis), 0.96 (6H, t, J=7.28 Hz, γ′′-H), 1.38 (6H, two doublets superimposed, J=6.52, 6.76 Hz, γ′-H), 1.64 (4H, pseudo-quintet, J=7.80, 7.52, 7.28 Hz, β′′-H), 2.55 (4H, pseudo-triplet, J= 7.28, 8.04 Hz, α′′-H), 3.44 (2H, m, β′-H), 3.55 (superimposed, β′-H-cis), 3.55-3.95 (m, γ-H), 3.84-3.89 (multiple singlets corresponding to ArOMe and overlapping γ-H from a mixture of erythro and threo 3 diastereoisomers as well as containing β′-H-*cis* hydrogen), 4.05 (m, β-H from threo diastereoisomer), 4.18 (broad m, β -H from erythro diastereoisomer), 4.98 (broad m, 2H, α-H), 5.09 (2H, two doublets superimposed, J=9.52 and 9.28 Hz, α′-H), 6.15 (four singlets, ArOH), 6.60 (d, 4H, Ar, J=14.28 Hz), 6.88-7.10 (m, 10H, Ar)

^{13}C NMR (100 MHz, $CDCl_3$) δC ppm- 13.93, 17.60, 25.10, 29.27, 38.09, 45.82, 55.97, 55.99, 56.21, 60.95, 72.87, 87.17, 92.96, 108.45, 110.33, 111.86, 115.43, 120.03,120.51, 121.22, 122.24, 124.12, 131.78, 132.97, 136.55, 142.16, 142.60, 143.87, 145.23, 146.81, 146.89, 147.44, 151.26, 151.53

MS calculated 1047.20, mass found [M+NH_4^+] = 1064.50

Values obtained are consistent with reported literature [2]

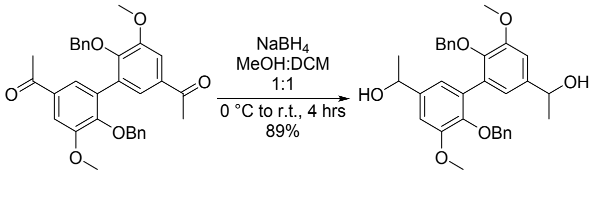

Benzyl acetovanillone (1.5 g, 2.94 mmol) added to flask and made into solution by addition of DCM and MeOH (20 mL each to total volume 40mL) and then flask cooled in ice-bath to 0 °C after which sodium borohydride (0.44 g, 11.8 mmol, 4 eq) was added and the reaction mixture was allowed to come to room temperature slowly. Reaction mixture allowed to stir until complete consumption of starting material observed by TLC with eluent 2:1 EtOAc: P.E anisaldehyde stain, when presence of slower moving spot was the dominant on the plate, the reaction was quenched by the addition of H_2O (35 mL) the addition of EtOAc (60 mL) allowed the separation of the organic and aqueous phase. The combined organic phases were dried over anhydrous magnesium sulphate, filtered, and concentrated *in vacuo* to provide a yellow solid which was carried forward without purification.

^{1}H NMR (400 MHz, $CDCl_3$) δH ppm 1.34 (6H, d, $J_{a\text{-}b}$= 6.32 Hz, 2 x CH_3), 1.96 (2H, bs, 2 x OH) 3.80 (6H, s, 2 x OMe), 4.67 (6H, m, overlapping CH_2Ar & CHOH), 6.76 (2H, m, Ar), 6.90 (2H, s, Ar), 6.97 (4H, m, Ar), 7.09 (6H, m, Ar)

^{13}C NMR (100 MHz, $CDCl_3$) δC ppm 23.94, 55.02, 69.14, 73.63, 107.93, 119.48, 126.41, 126.84, 126.93, 131.62, 131.64, 136.92, 140.18, 140.20, 144.06, 152.13

MS calculated 514.62, mass found [M+H$^+$] = 537.23

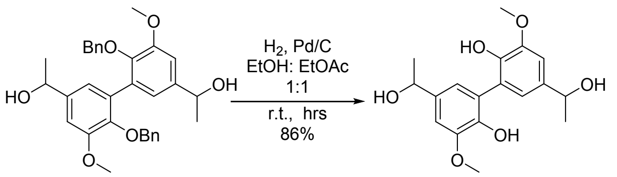

Flask charged with benzylated alcohol species (0.98 g, 1.91 mmol) and made into solution by addition of EtOH and EtOAc (15 mL each to total 30 mL), flask was then placed under N_2 by purging 8 times under vacuum with N_2, to yellowish solution was then added palladium on carbon (98 mg) and then flask was then placed under vacuum whereby it was purged 8 times with atmospheric hydrogen from a balloon. The reaction mixture was allowed to stir under positive pressure of hydrogen until all the starting material was consumed, this was monitored by TLC with eluent 3:1 EtOAc: P.E anisaldehyde stain. When the formation of the slower moving spot was the dominant and the faster moving spot was removed the reaction was stopped by filtering the solution slowly through celite under light vacuum. The filtrate was stripped down under reduced pressure whereafter the oil produced was chromatographed on silica gel with eluent 1:1 EtOAc: P.E to 2:1 EtOAc: P.E, to provide the product as an off-brown oil in 86% yield.

^{1}H NMR (400 MHz, $CDCl_3$) δH ppm 1.51 (6H, d, $J_{a\text{-}b}$= 6.38 Hz, 2 X CH_3), 3.94 (6H, s, 2 x OMe), 4.86 (2H, q, $J_{a\text{-}b}$= 6.36 Hz, $J_{a\text{-}a'}$= 12.86 Hz, 2 x CHOH), 6.17 (2H, bs, 2 x ArOH), 6.90 (2H, m, Ar), 6.97 (2H, m, Ar)

^{13}C NMR (100 MHz, $CDCl_3$) δC ppm 25.03, 56.16, 70.37, 107.58, 120.37, 124.07, 137.84, 142.04, 147.36

MS calculated 334.37, mass found [M+H$^+$] = 668.74

LC-MS chromatograms for reaction intermediate products

Products

P1

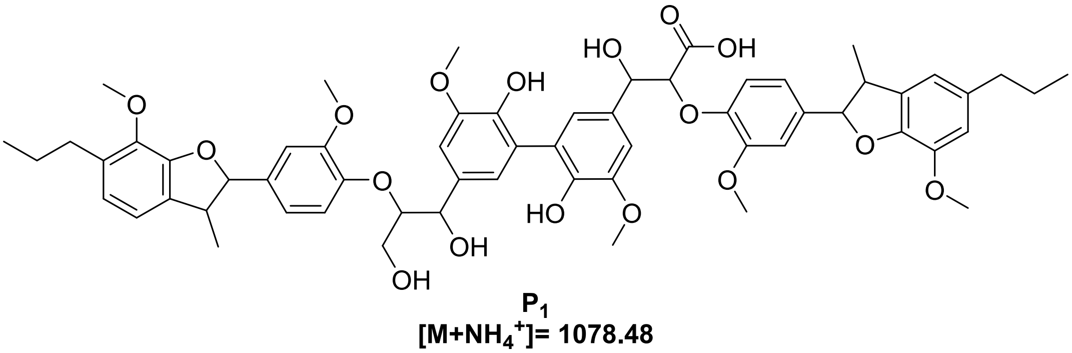

P_1
$[M+NH_4^+]$= 1078.48

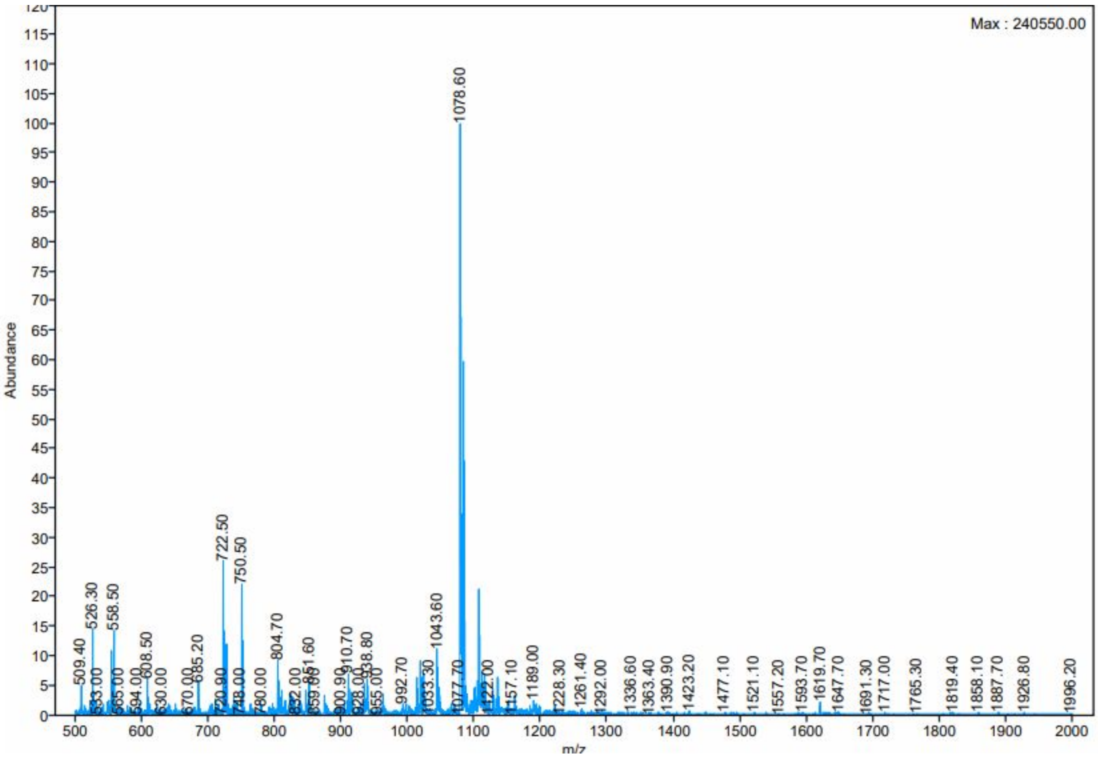

Figure S3. Structure and associated mass spectrum of intermediate **P_1**

P2

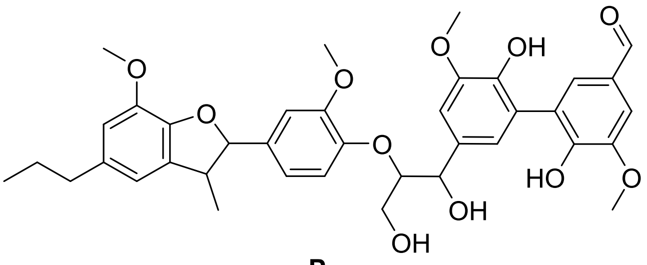

P_2
$[M+NH_4^+]$= 692.31

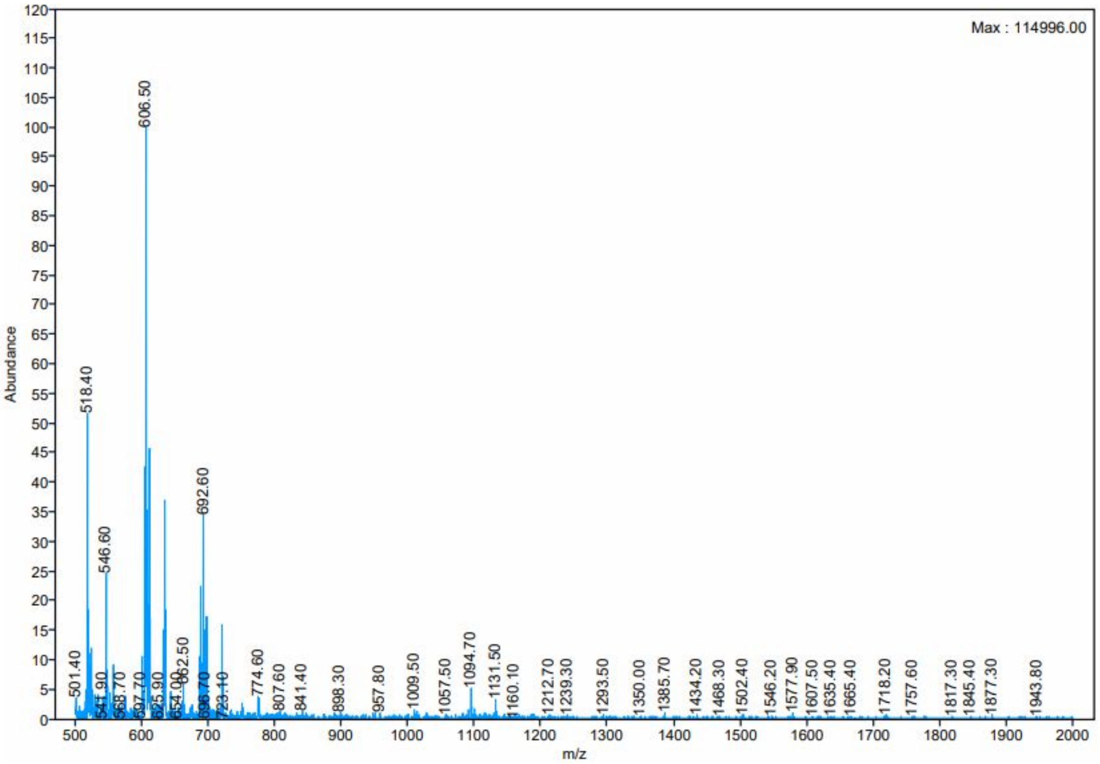

Figure S4. Structure and associated mass spectrum of intermediate **P_2**

P3

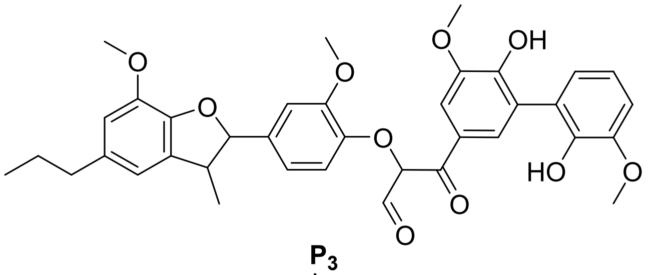

P_3
$[M+NH_4^+]$= 660.28

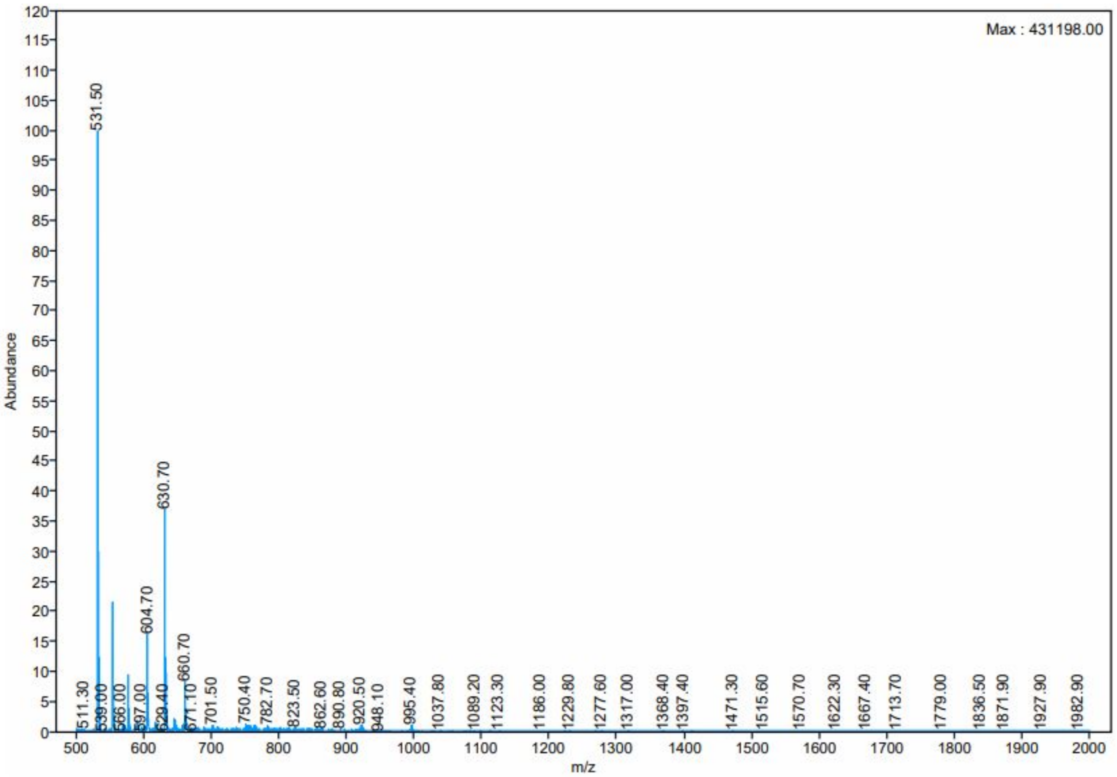

Figure S5. Structure and associated mass spectrum of intermediate **P_3**

P4

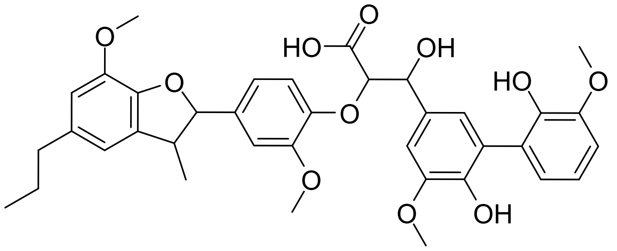

P_4
$[M+H^+]$= 661.26
$[M+Na^+]$= 683.25
$[2M+Na^+]$= 1343.50

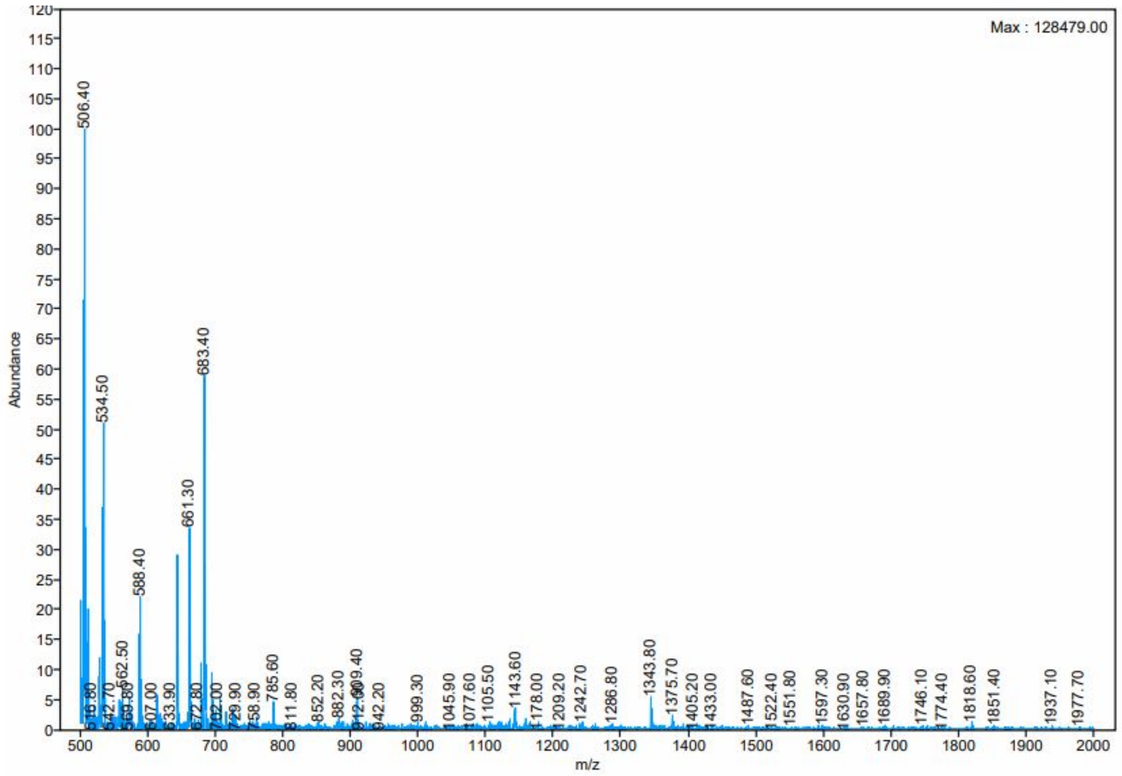

Figure S6. Structure and associated mass spectrum of intermediate **P_4**

P5

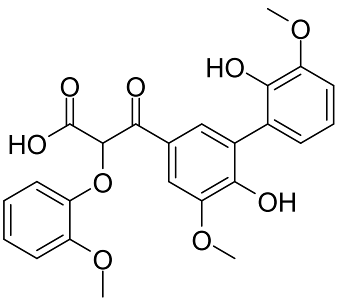

P_5
$[2M+H^+]$= 909.26

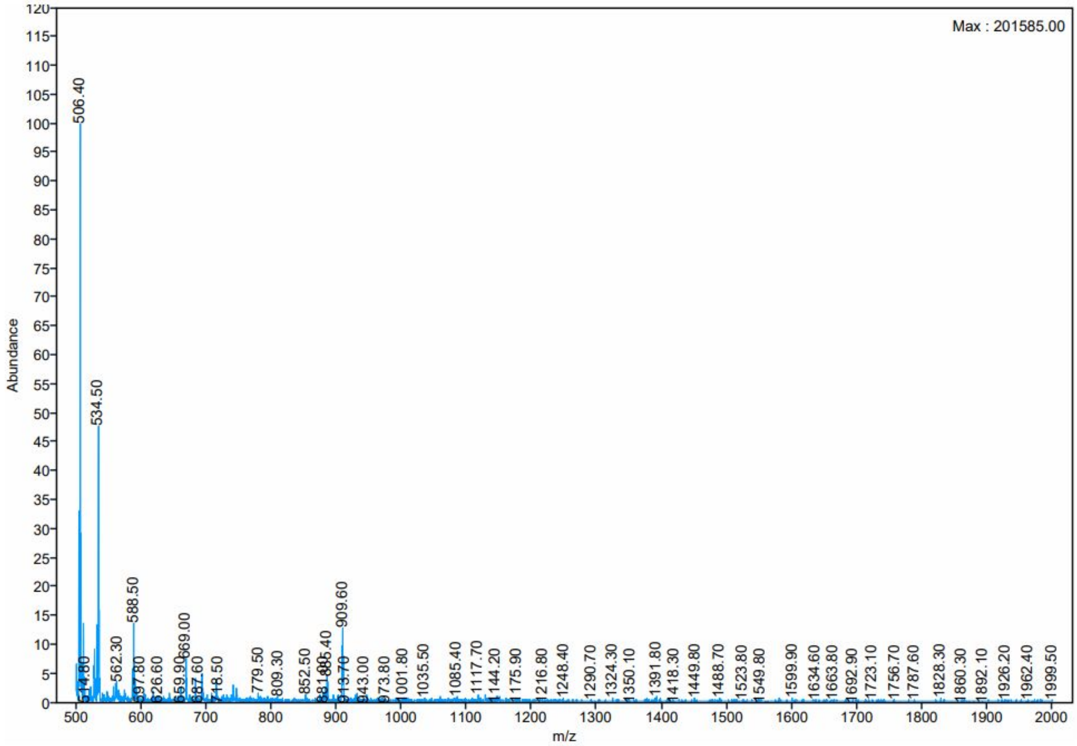

Figure S7. Structure and associated mass spectrum of intermediate **P_5**

P6

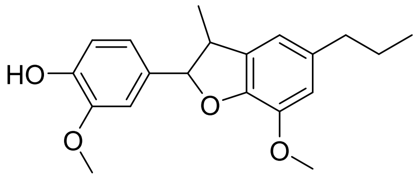

P_6
Mass found:
$[2M+NH_4^+]$= 674.37

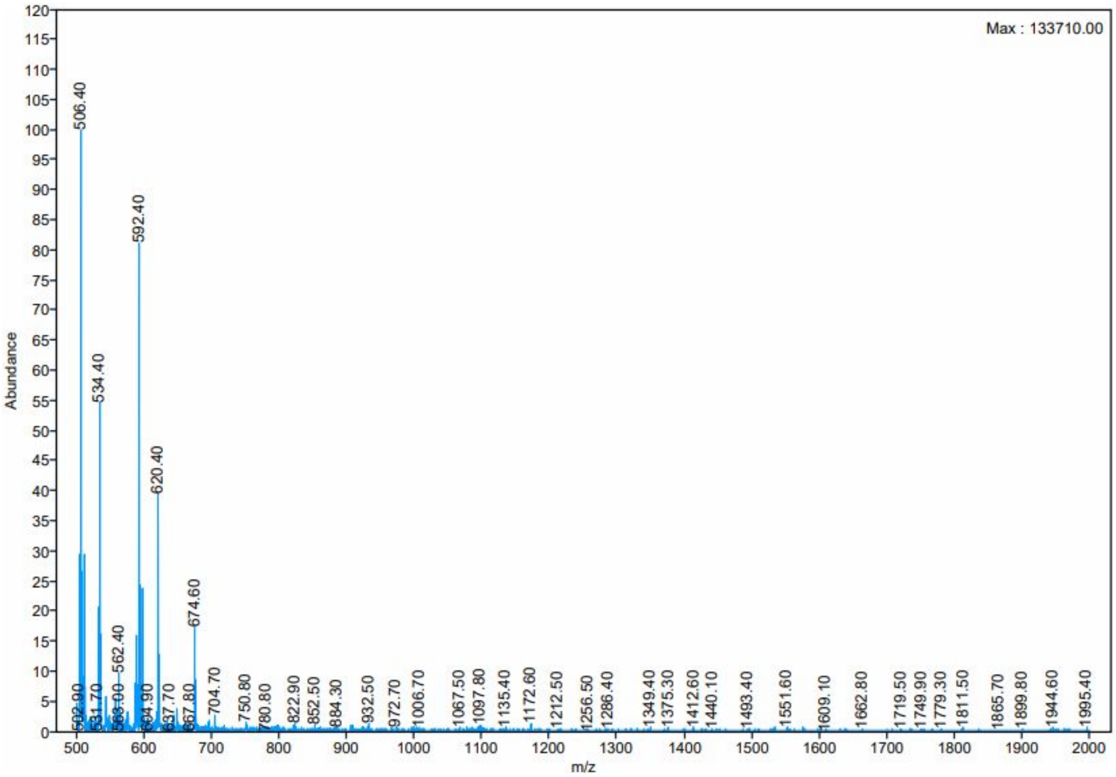

Figure S8. Structure and associated mass spectrum of intermediate **P_6**

P7

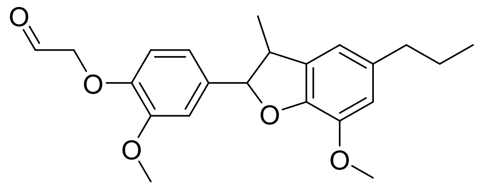

P_7
$[2M+NH_4^+]$= 758.39

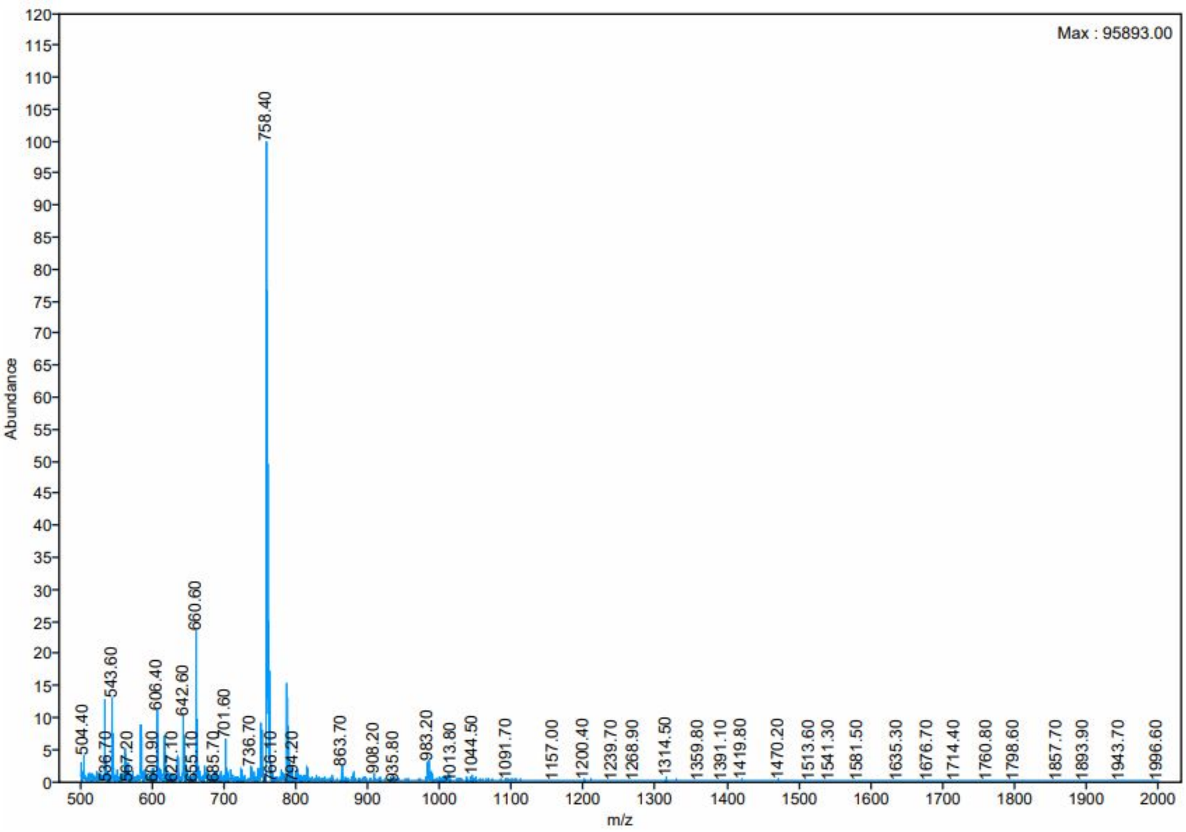

Figure S9. Structure and associated mass spectrum of intermediate **P_7**

P8

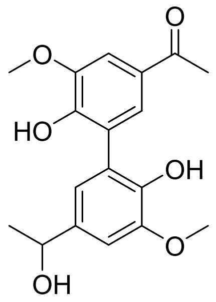

P_8
[$2M+NH_4^+$]= 682.29

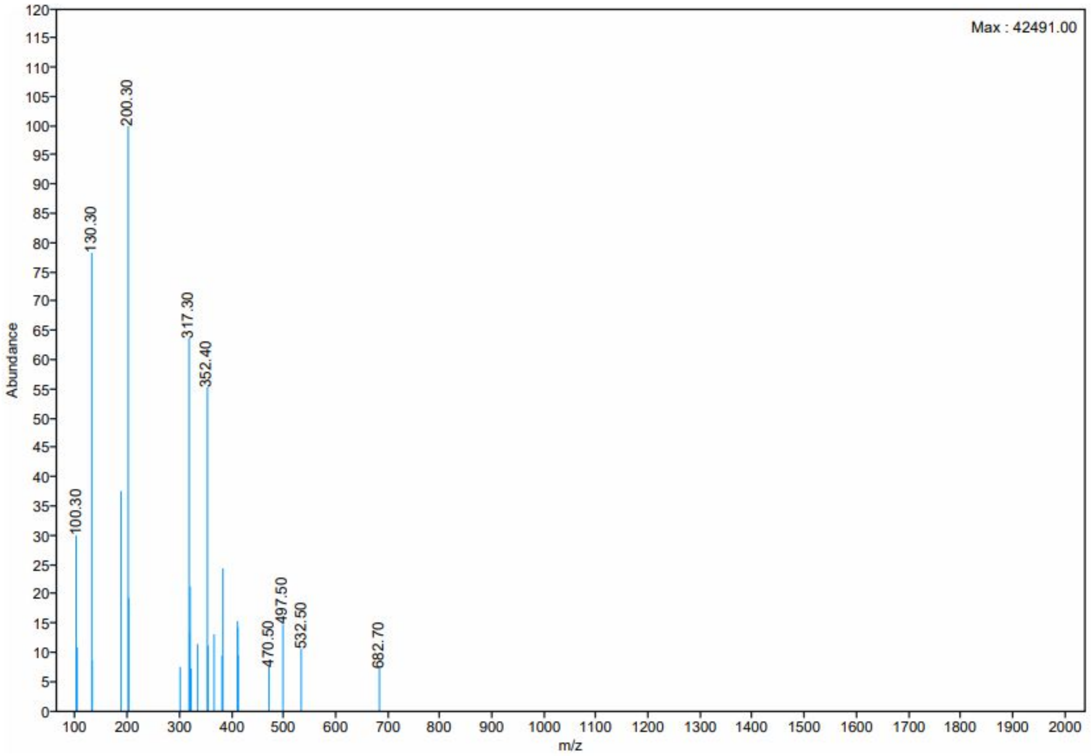

Figure S10. Structure and associated mass spectrum of intermediate **P_8**

P9

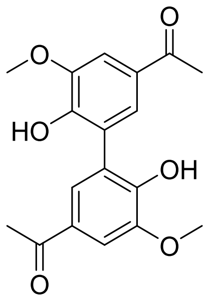

P_9
$[M+H^+]$= 331.12
$[2M+H^+]$= 661.23

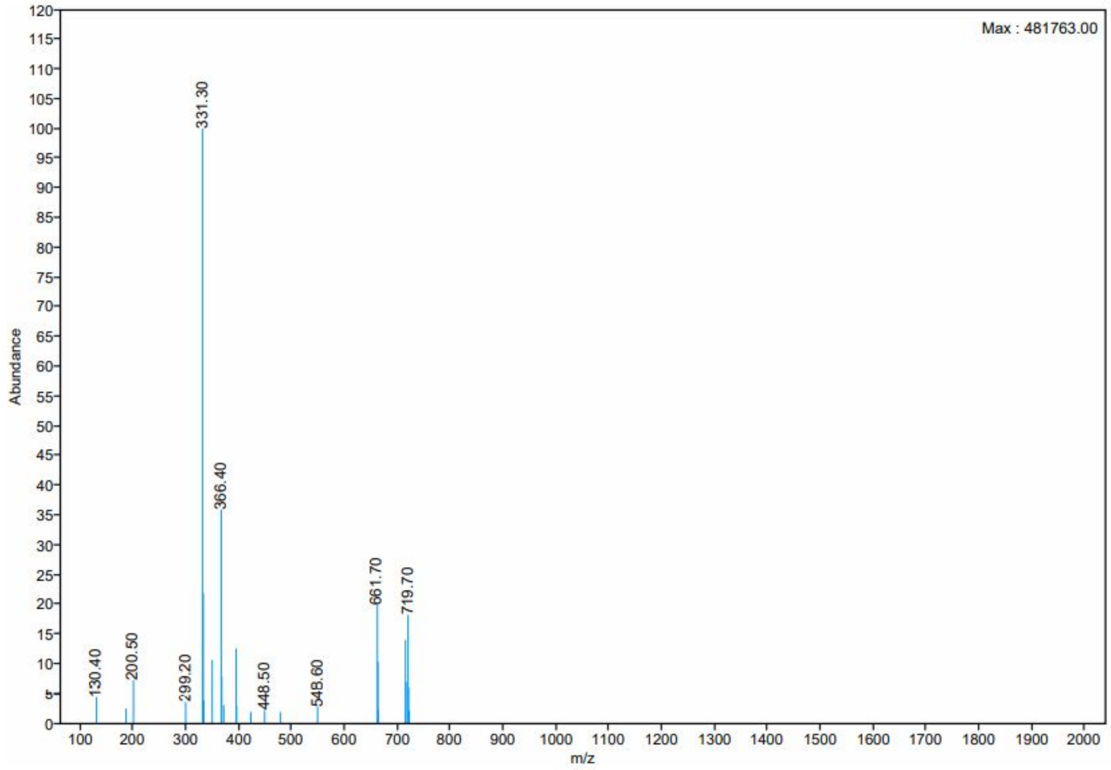

Figure S11. Structure and associated mass spectrum of intermediate **P_9**

P10

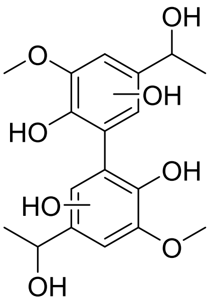

P_{10}
[2M+Na⁺]= 755.25

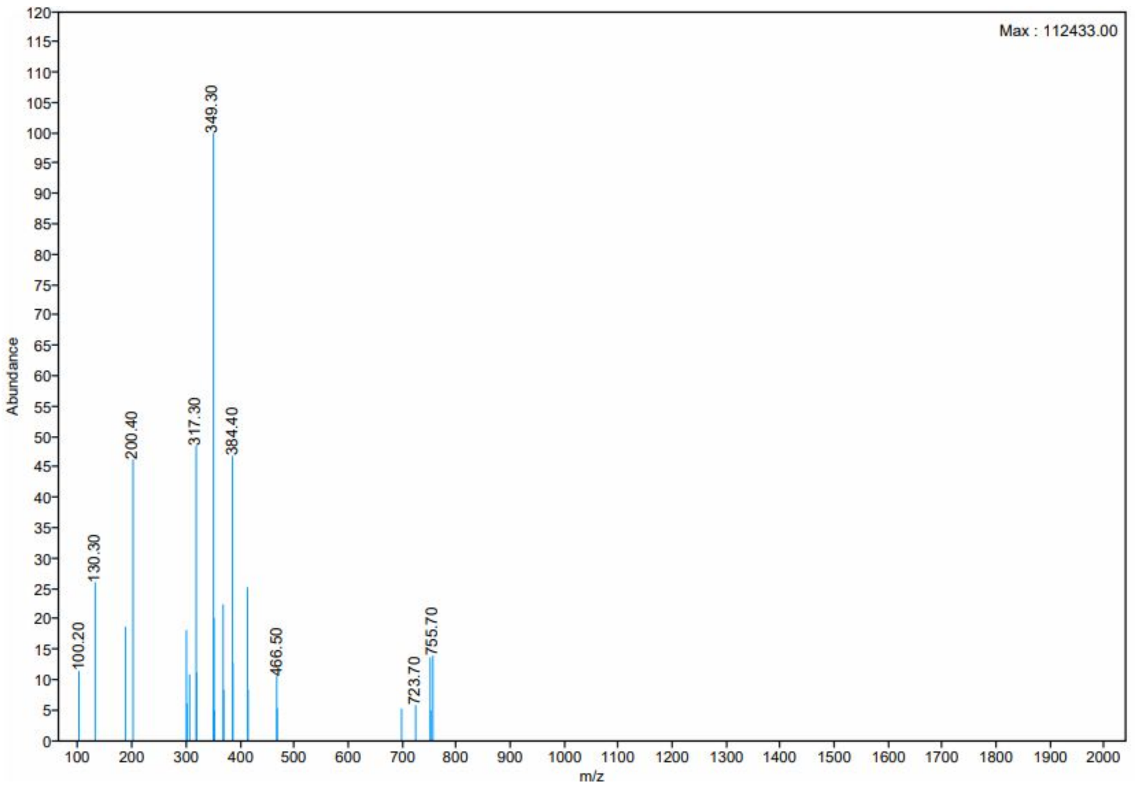

Figure S12. Structure and associated mass spectrum of intermediate $\mathbf{P_{10}}$

Results

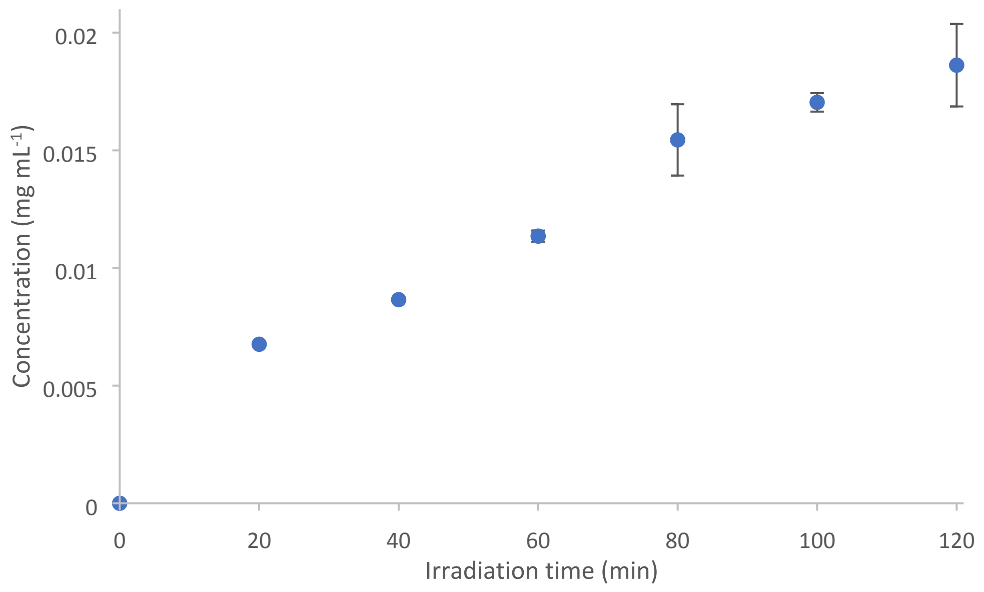

Figure S13: Generation of reaction intermediate P6 (b-5 model dimer) during the photocatalytic conversion of the hexameric substrate

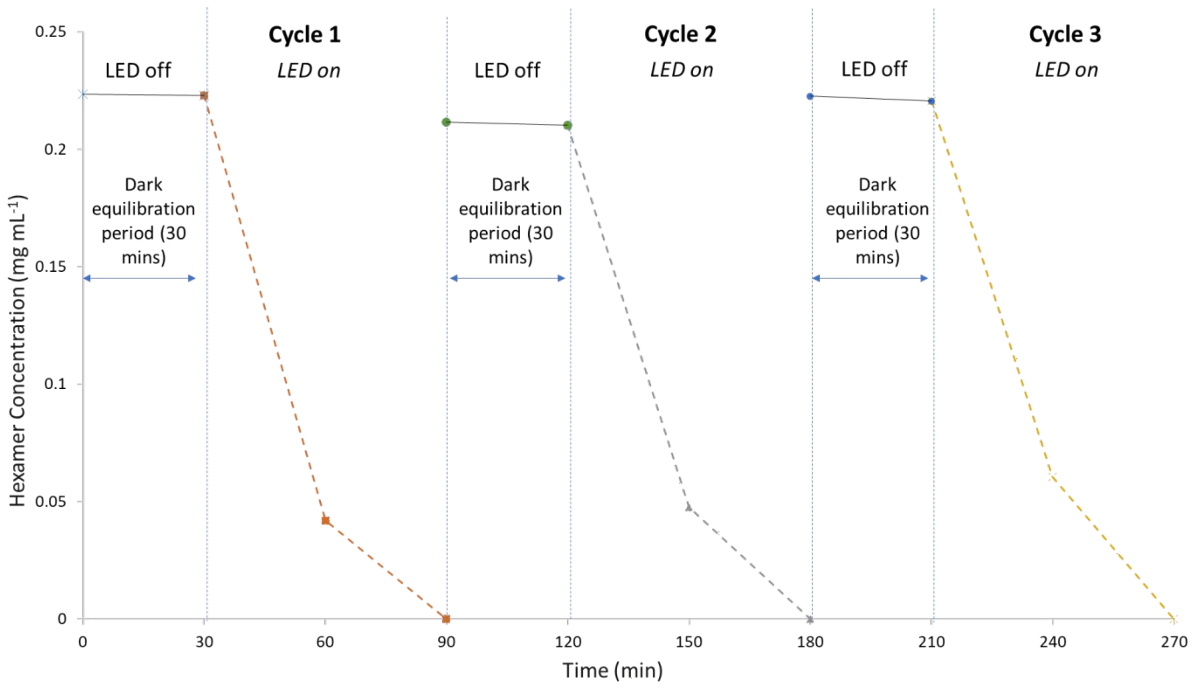

Figure S14: Activity of the TiO_2 photocatalyst over 3 reaction cycles. Fresh hexamer substrate and reaction solvent were added between each cycle. Trendlines are added into each cycle for clarity.
